# High-performance compliant thermoelectric generators with magnetically self-assembled soft heat conductors for self-powered wearable electronics

**DOI:** 10.1038/s41467-020-19756-z

**Published:** 2020-11-23

**Authors:** Byeongmoon Lee, Hyeon Cho, Kyung Tae Park, Jin-Sang Kim, Min Park, Heesuk Kim, Yongtaek Hong, Seungjun Chung

**Affiliations:** 1 Department of Electronic and Computer Engineering, Inter-University Semiconductor Research Center (ISRC), Seoul National University, Seoul, 08826 Korea; 2 Soft Hybrid Materials Research Center, Korea Institute of Science and Technology, Seoul, 02792 Korea; 3 Institute of Advanced Composite Materials, Korea Institute of Science and Technology, Wanju, Jeonbuk 55324 Korea; 4 KHU-KIST Department of Converging Science and Technology, Kyung Hee University, Seoul, 02447 Korea; 5 Present Address: Soft Hybrid Materials Research Center, Korea Institute of Science and Technology, Seoul, 02792 Korea

**Keywords:** Devices for energy harvesting, Thermoelectric devices and materials, Electrical and electronic engineering

## Abstract

Softening of thermoelectric generators facilitates conformal contact with arbitrary-shaped heat sources, which offers an opportunity to realize self-powered wearable applications. However, existing wearable thermoelectric devices inevitably exhibit reduced thermoelectric conversion efficiency due to the parasitic heat loss in high-thermal-impedance polymer substrates and poor thermal contact arising from rigid interconnects. Here, we propose compliant thermoelectric generators with intrinsically stretchable interconnects and soft heat conductors that achieve high thermoelectric performance and unprecedented conformability simultaneously. The silver-nanowire-based soft electrodes interconnect bismuth-telluride-based thermoelectric legs, effectively absorbing strain energy, which allows our thermoelectric generators to conform perfectly to curved surfaces. Metal particles magnetically self-assembled in elastomeric substrates form soft heat conductors that significantly enhance the heat transfer to the thermoelectric legs, thereby maximizing energy conversion efficiency on three-dimensional heat sources. Moreover, automated additive manufacturing paves the way for realizing self-powered wearable applications comprising hundreds of thermoelectric legs with high customizability under ambient conditions.

## Abstract (web summary)

Though flexible thermoelectric generators (TEGs) are attractive for energy harvesting applications, existing devices show low efficiency due to heat loss and poor thermal contact. Here, the authors report high-performance conformable TEGs with stretchable interconnects and soft heat conductors.

## Introduction

As the “Internet of Things” (IoT) enables all objects to be wirelessly interconnected, one of the long-standing demands is the realization of self-powered electronics that do not require additional power sources or batteries^1–5^. Thermoelectric generators (TEGs) have been regarded as one of the most promising candidates for independent energy harvesting due to their ability to convert waste heat to usable electricity^6–13^. According to ${\it{P}} = \frac{{\eta _{\mathrm{o}}\left( {zT} \right)}}{{T_{\mathrm{H}}}}{\Delta} T_{{\mathrm{TE}}}Q_{{\mathrm{TE}}}$, where η_o_, *T*_H_, Δ*T*_TE_, and *Q*_TE_ are the energy conversion efficiency depending on the figure of merit (*zT*) of thermoelectric (TE) materials^14–17^, the hot-side temperature, the temperature difference across TE legs, and the heat flow through the TE legs, respectively, Δ*T*_TE_ is the most important factor for given thermal energy and TE material properties^18–20^. In other words, minimization of the parasitic heat loss from conformal contact^21–23^ and a low thermal impedance between TE materials and heat sources^24–27^ are key to achieving a high energy conversion efficiency that can accelerate the realization of practical self-powered applications.

From the perspective of high-performance TE materials, the most favorable candidates have been bismuth telluride (Bi_2_Te_3_) alloys^27–30^. For the last decade, beyond traditional Bi_2_Te_3_-based rigid TEGs, which allow thermal energy harvesting only from planar-shaped heat sources^24,30,31^, efforts to utilize these alloys in realizing flexible TEGs that facilitate thermal energy harvesting on three-dimensional (3D) heat sources have been made^10,27,32–42^. As a representative approach, the infiltration of a soft medium, e.g., polydimethylsiloxane (PDMS), provides acceptable mechanical tolerance to inorganic TEGs^10,35,36,39,42^. However, the previous results showed unexpectedly poor TE performances due to the parasitic heat loss in high-thermal-impedance polymer substrates, undesirable air gaps arising from limited mechanical conformability, and low device yield of manual manufacturing. Therefore, implementation of high-performance TEGs that can conform perfectly to arbitrary-shaped heat sources with enhanced heat transfer ability via straightforward manufacturing is highly desirable.

Here, we report compliant TEGs simultaneously exhibiting an enhanced heat exchange capability and unprecedented conformability via soft heat conductors (s-HCs) and intrinsically stretchable interconnects, respectively, which facilitate efficient thermal energy harvesting from arbitrary-shaped heat sources. The magnetically self-assembled silver-coated nickel (Ag–Ni) particles in a PDMS matrix with a thermal conductivity of ~1.4 W m^−1^ K^−1^ are lined up with Bi_2_Te_3_ bulk legs, which significantly improves the heat transfer ability in the through-plane direction, achieving the minimized parasitic heat loss and conformal thermal contact. The silver-nanowire (AgNW)-based stretchable interconnects allow stretchability up to 20%, enabling our TEG to conform perfectly to curved surfaces without air gaps that cause significant heat loss. The highly automated manufacturing procedure enables the implementation of a compliant TEG comprising 220-np-pairs exhibiting a maximum power of 7.02 mW and an open-circuit voltage (*V*_OC_) of 2.12 V at a given temperature difference (Δ*T*_Applied_) of 40 K, which is integrated with a flexible circuit to demonstrate self-powered wearable warning systems indicating an abrupt temperature increase with light-emitting alarms.

## Results

### Design of and highly automated process for compliant TEGs

**Fig. 1:**
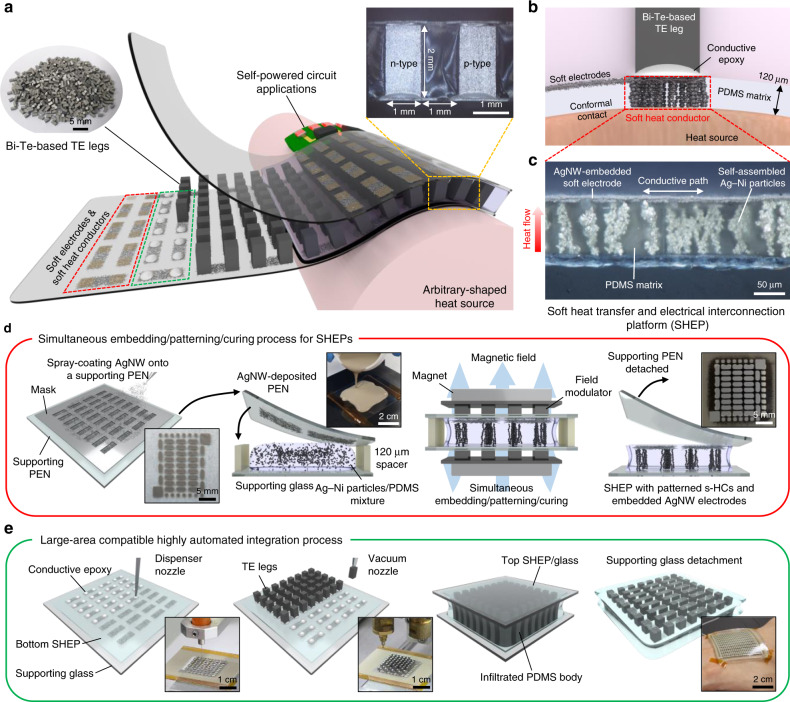
Design and fabrication process for compliant thermoelectric generator (TEGs). **a** Conceptual illustration of a compliant TEG with soft electrodes and soft heat conductors (s-HCs) for self-powered circuit applications. The left inset is a photograph of bismuth telluride (Bi_2_Te_3_)-based thermoelectric (TE) legs and the right inset is an optical image of a cross-section of the compliant TEG. Scale bars, 5 and 1 mm. **b** Schematic illustration showing the structure of the compliant TEG establishing conformal contact with an arbitrary-shaped heat source. The s-HCs efficiently transfer heat energy from the heat source to the TE legs, and the soft electrodes allow a high degree of mechanical freedom. **c** Microscopic image of the soft heat transfer and electrical interconnection platform (SHEP) with embedded soft silver nanowire (AgNW) electrodes and patterned silver-coated nickel (Ag–Ni) s-HCs. Scale bar, 50 μm. **d** Schematic illustration and photographs of simultaneous embedding/patterning/curing process for the SHEPs. Scale bars, 5 mm, 2 cm, and 5 mm. **e** Schematic illustration and photographs of a large-area compatible fully automated integration process using a programmable dispenser and a pick-and-place machine. The rightmost photograph shows a compliant TEG comprising 440 TE legs conformably attached to human skin. Scale bars, 1 cm.

Figure 1a illustrates the concept and design of our compliant TEGs. Previously reported high-performance compliant TEGs have generally employed thick and rigid electrodes to interconnect the TE legs^35,36,39^ and suffered from limited mechanical flexibility and complicated fabrication processes. Liquid metals, such as eutectic gallium–indium (EGaIn), have been used for soft interconnects^37,38,42^, but their deleterious and unstable nature requires high-thermal-impedance polymer encapsulation, significantly impeding heat transfer to TE materials. To address these issues, we develop a soft heat transfer and electrical interconnection platform (SHEP) in which intrinsically stretchable electrodes and s-HCs are embedded, which interconnect high-*zT* TE legs and interface them with arbitrary-shaped heat sources, respectively, while maintaining both mechanical softness and a low thermal impedance (Fig. 1b, c). The soft electrodes and s-HCs were facilely formed in an elastomer matrix by a simultaneous embedding/patterning/curing process as follows (Fig. 1d and details in the “Methods”, Supplementary Fig. 1, Supplementary Table 1, and Supplementary Movie 1). We first covered a Ag–Ni particles/PDMS precursor mixture on a supporting glass by a AgNW-deposited polyethylene naphthalate (PEN) substrate. Then, we sandwiched them with two iron pillar arrays and attached two magnets at the top and bottom of the pillar arrays. Because the magnetic field is concentrated on the vertically aligned iron pillar couples, as shown in the finite element analysis (FEA) result in Supplementary Fig. 2, Ag–Ni particles rapidly converge into the location of the iron pillars. At the same time, the particles between the upper and lower pillars are self-assembled, forming well-defined vertical chains, i.e., percolation paths, in the PDMS mixture. After curing the mixture and detaching the PEN substrate, a SHEP on the supporting glass was produced. The SHEP formation process is fairly simple, highly customizable, and reproducible. The design of s-HC patterns can be facilely modulated by employing different iron pillar arrays. The Ag–Ni particle concentration in the s-HC patterns that determines the heat transfer ability can be easily adjusted by changing the volume fraction of the Ag–Ni particle/PDMS mixture (Supplementary Fig. 3). Furthermore, the potential parameters that affect the TE performance, such as magnetic flux intensity and PDMS viscosity, were elaborately optimized to be in the middle of the process window, rendering our process more stable and reliable under ambient conditions (Supplementary Figs. 4 and 5). The Bi_2_Te_3_-based TE legs were then integrated onto the prepared SHEPs by fully automated epoxy printing and pick-and-place processes (Fig. 1e). After attaching top SHEP onto the TE leg array and infiltrating PDMS between the top and bottom SHEPs for further improving mechanical robustness, a compliant TEG with intrinsically stretchable electrodes and s-HCs was finally produced. The total process time is ~4.5 h, comprising 2 h for the SHEP formation and 2.5 h for the integration.

Our manufacturing strategy is in line with the accelerating trends in miniaturization for wearable applications. In the past years, bulk legs have been manually placed onto polymer-based substrates, which limits the degree of integration, or fill factor (FF), closely related to the power density in wearable applications^4,18,25^. In contrast, our highly automated integration offers a high degree of scalability and customizability along with a high device yield, which allows the reliable implementation of large-area compliant TEGs with even 440 TE legs in an area of 3.9 × 4.3 cm^2^.

### Thermal and mechanical characterization of the s-HCs

**Fig. 2:**
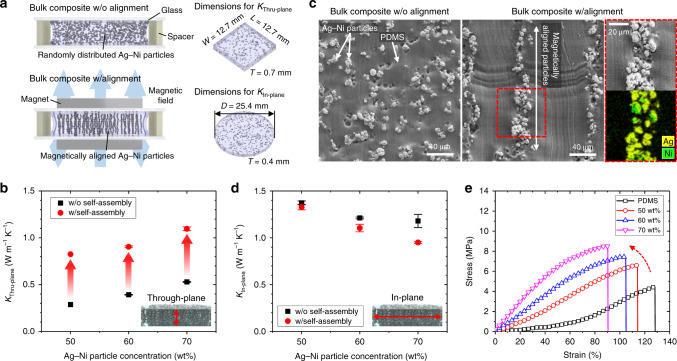
Thermal and mechanical properties of s-HCs. **a** Schematic illustration of bulk Ag–Ni particle/polydimethylsiloxane (PDMS) composites without and with magnetic self-assembly for measuring the through-plane and in-plane thermal conductivity. **b** Through-plane thermal conductivity as a function of Ag–Ni particle concentration without and with magnetic self-assembly. The error bars represent the standard deviation. **c** Scanning electron microscope (SEM) images of bulk composites without and with magnetic self-assembly. A magnified SEM image and an energy dispersive spectrometry (EDS) image showing the vertically aligned Ag–Ni particles are also included. Scale bars, 40 μm. **d** In-plane thermal conductivity as a function of Ag–Ni particle concentration without and with magnetic self-assembly. The error bars represent the standard deviation. **e** Strain–stress curves of bare PDMS and magnetically self-assembled Ag–Ni particle/PDMS composites with different Ag–Ni particle concentrations.

To investigate the effect of our s-HCs on the heat transfer ability of elastomeric substrates, we systematically analyzed the thermal conductivity of bulk Ag–Ni particle/PDMS composites, i.e., composites not spatially patterned by the iron pillar arrays, as a function of the Ag–Ni particle concentration with and without magnetic self-assembly (Fig. 2a). The through-plane thermal conductivity (*K*_Thru-plane_) of the composites increased from 0.15 to 0.53 W m^−1^ K^−1^ as the Ag–Ni particle concentration increased to 70 wt% and drastically jumped to 1.1 W m^−1^ K^−1^ upon magnetic self-assembly (Fig. 2b). This result is primarily due to the greatly increased number of vertical percolation paths arising from the applied magnetic field, as shown in the scanning electron microscopy (SEM) images in Fig. 2c and Supplementary Fig. 6. Figure 2d shows the in-plane thermal conductivity (*K*_In-plane_) of the bulk composites. The composites without magnetic self-assembly showed *K*_In-plane_ > 1 W m^−1^ K^−1^, which is much higher than *K*_Thru-plane_; this result could be attributed to the inhomogeneous Ag–Ni particle distribution in the vertical direction due to the force of gravity during the curing process. After magnetic self-assembly, as high-density Ag–Ni particles at the bottom of the composite participated in the vertical chains along the direction of the magnetic field, *K*_In-plane_ slightly decreased. Notably, our strategy exploiting magnetic self-assembly of Ag–Ni particles effectively improved *K*_Thru-plane_, which is closely related to the ability to transfer heat to the TE legs, without a significant loss in *K*_In-plane_.

Furthermore, because the s-HCs were spatially patterned in a 1.2 × 1.2 mm^2^ square by using an iron pillar array, the estimated Ag–Ni particle concentration in the patterned s-HCs was ~85 wt% (Supplementary Fig. 3 and Supplementary Note 1 for details). The accurate thermal conductivity of our patterned s-HCs could not be experimentally obtained due to the limitation of our measurement system in terms of minimum dimensions and the difficulty in fabricating a bulk composite containing >75 wt% Ag–Ni particles. Therefore, a *K*_Thru-plane_ of ~1.4 W m^−1^ K^−1^ for an Ag–Ni particle concentration of 85 wt% was extracted from the extrapolation of the measured data in Fig. 2b. This thermal conductivity is comparable to that of a Bi_2_Te_3_ leg (~1.9 W m^−1^ K^−1^, see Supplementary Table 2) and results in a well-matched thermal impedance, significantly reducing the parasitic heat loss across the elastomeric layer to the linear scale. In addition, our s-HCs maintained their softness well, with a Young’s modulus <10 MPa and a fracture strain >90% (Fig. 2e), exhibiting better stretchability than most of the reference commercial thermal pads (Supplementary Fig. 7). These results indicate that the heat transfer ability of our s-HC is superior to those of previously reported compliant substrates (Supplementary Table 3), such as PDMS and engineered Ecoflex, and even comparable to those of commercial thermal pads.

### Enhanced TE performance via s-HCs

**Fig. 3:**
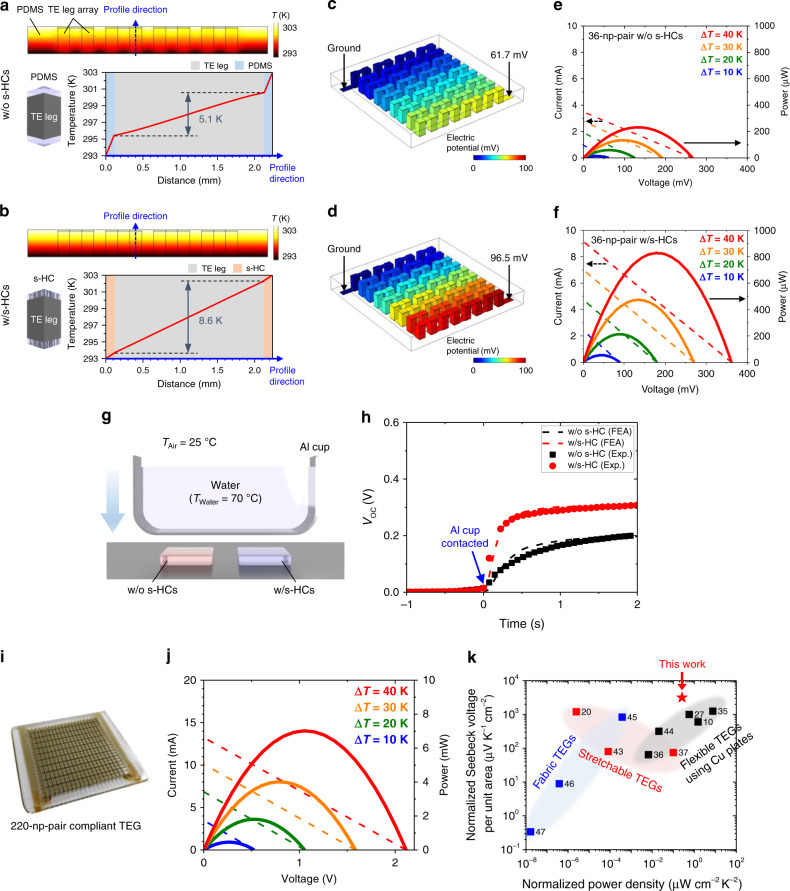
Effect of s-HCs on the TE performance of the compliant TEG. **a**, **b** Finite element analysis (FEA) results showing the temperature distribution on the cross-section of the TEGs without (**a**) and with s-HCs (**b**) for a given temperature difference of 10 K. **c**, **d** FEA results showing the open-circuit voltage (*V*_OC_) of the 36-np-pair TEGs without (**c**) and with s-HCs (**d**) for a given temperature difference of 10 K. **e**, **f** Experimentally measured TE performances of 36-np-pair TEGs without (**e**) and with s-HCs (**f**) showing current and power as a function of voltage. **g** Schematic illustration of the experimental setup for measuring the response of the TEGs without and with s-HCs. **h** Time-resolved *V*_OC_ of the two TEGs when the aluminum cup with hot water is abruptly placed in contact with the two TEGs. The dashed lines represent the corresponding FEA results. **i** Photograph showing the 220-np-pair-compliant TEG. **j** Experimentally measured TE performance of the 220-np-pair-compliant TEG including current and power as a function of voltage. **k** Performance comparison of wearable TEGs.

To investigate the effect of our s-HC on the power generation, we characterized the performances of 36-np-pair TEGs with and without s-HCs by both 3D FEA and experimental measurements. Figure 3a, b presents the FEA results showing the temperature distribution on a cross-section of the two TEGs when the Δ*T*_Applied_ between the top and bottom boundaries was 10 K (modeling details in Supplementary Fig. 8 and Supplementary Table 4). In the case of the TEG without s-HCs, although the PDMS supporting layers were sufficiently thin (120 µm) compared to the height of the TE leg, the Δ*T*_TE_ was only 5.1 K (Fig. 3a). The main reason for this small difference is that the thermal conductivity of PDMS (~0.16 W m^−1^ K^−1^) is extremely low compared to that of the TE leg; therefore, a large portion of Δ*T*_Applied_ was lost across the PDMS layers. In contrast, the temperature was linearly distributed across the TEG with s-HCs, showing a Δ*T*_TE_ of 8.6 K, as a result of the thermal impedance matching between the s-HCs and TE leg due to the drastically improved *K*_Thru-plane_ with the s-HCs (Fig. 3b). The resulting *V*_OC_ values calculated from the FEA were 61.7 and 96.5 mV for the TEGs without and with s-HCs, respectively (Fig. 3c, d). We also calculated *V*_OC_ as a function of the thermal conductivity of the s-HC, showing that our s-HC enables ~86% of the maximum theoretical *V*_OC_ for the 36-np-pair TE legs to be reached (Supplementary Fig. 9). This effect of the s-HCs was also verified by experimentally measuring the TE performances of the fabricated 36-np-pair-compliant TEGs without and with s-HCs using homemade measurement equipment (Fig. 3e, f and Supplementary Fig. 10). Both TEGs showed linear and quadratic increases in *V*_OC_ and the output power, respectively, as Δ*T*_Applied_ increased. The TEG with s-HCs showed 45% higher *V*_OC_ compared to that without s-HCs, 61.4 versus 89.5 mV at a Δ*T*_Applied_ of 10 K, which is consistent with the FEA results. The maximum powers of the TEGs without and with s-HCs at a Δ*T*_Applied_ of 40 K were 232 and 828 μW, respectively. Although the *V*_OC_ can be further improved by increasing the thermal conductivity of the s-HC or decreasing the thickness of the s-HC, the parameters were optimized with regard to softness, mechanical reliability, and process stability. Furthermore, the improved heat transfer ability of our compliant TEG allows a fast response to a dynamic temperature change. We measured the time-resolved *V*_OC_ of the two TEGs on an aluminum plate when an aluminum cup with hot water (~70 °C) was abruptly placed in contact with their top surfaces (Fig. 3g and Supplementary Fig. 11). The TEG with s-HCs responded to the temperature change faster than that without s-HCs, with a higher maximum *V*_OC_, showing good agreement with the FEA results (Fig. 3h).

A long-term demand of wearable TEG applications is reliable operation of high-FF compliant TEGs. Using our scalable and automated fabrication process, we fabricated a compliant TEG with 440 TE legs in an area of 3.9 × 4.3 cm^2^ (Fig. 3i). The TEG generated a maximum power of 7.02 mW and a *V*_OC_ of 2.12 V at a Δ*T*_Applied_ of 40 K (Fig. 3j). Figure 3k and Supplementary Table 3 show the performance comparison between the state-of-the-art flexible/wearable TEGs^10,20,27,35–37,43–47^. Our compliant TEG with the s-HCs shows the highest normalized Seebeck voltage per unit area, the *V*_OC_ normalized by the Δ*T*, and the dimension of TEGs, due to the outstanding heat transfer ability of the s-HCs and high FF achieved by the automated integration process. Furthermore, it shows the highest normalized power density, the power density divided by Δ*T*^2^ to exclude the Δ*T* dependency, in the stretchable TEG group. The value of 0.26 μW cm^−2^ K^−2^ is even comparable to the top records of previous flexible TEGs with the rigid Cu electrodes, although some of them show higher value >1 μW cm^−2^ K^−2^ owing to the high conductivity of the rigid Cu electrodes that severely compromise the conformability of TEGs. Especially, together with the excellent conformability, our TEG attached to human skin generated the highest maximum power density (6.96 μW cm^−2^) and *V*_OC_ (266 mV) in previously reported wearable TEGs on human skin (Supplementary Fig. 12).

The output power of our compliant TEG could be further improved by reducing the module resistance that is composed of the resistances of TE legs, AgNW-based stretchable electrodes, and conductive epoxy junctions between them (Supplementary Fig. 13). The “junction resistance” and “electrode resistance” account for 77% and 19% of the total module resistance, respectively (Supplementary Table 5). Therefore, the minimization of the resistance of both components is key to improving the output power of our compliant TEG. More specifically, the junction resistance can be again divided into three parts: a contact resistance between Ag epoxy and TE legs, bulk resistance of conductive epoxy, and contact resistance between conductive epoxy and AgNW electrode (Supplementary Fig. 14). Because the stretchable electrodes are based on a composite material where a AgNW random network is embedded in a PDMS matrix, a large portion of the surface is made up of a PDMS matrix where the AgNWs are partially exposed. This low density of surface-exposed conductive materials increases contact resistance between the electrodes and conductive epoxy (Supplementary Fig. 15). Feasible solutions could be partially etching the PDMS to expose AgNWs or using an interfacial layer such as a printed metal thin film between the conductive epoxy and stretchable electrodes. The length, diameter, and amount of the deposited AgNWs are also crucial parameters for the conductivity of the stretchable electrodes and contact resistance between the conductive epoxy and electrodes.

### Mechanical reliability of the compliant TEG

**Fig. 4:**
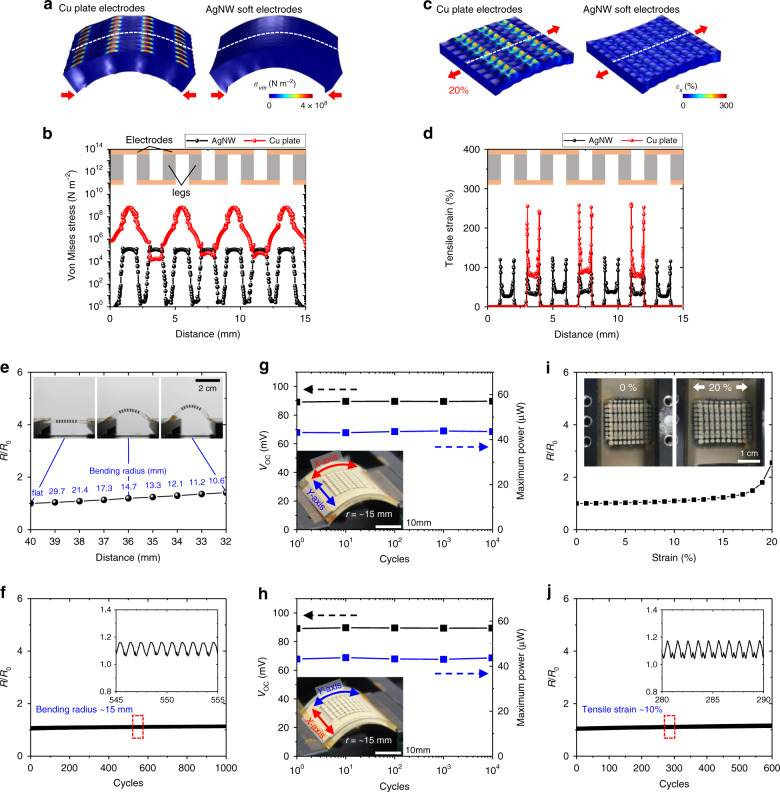
Mechanical reliability of the compliant TEG. **a** FEA results showing the von Mises stress of the surfaces of a TEG with Cu plate electrodes and a TEG with AgNW-embedded soft electrodes under bending conditions. **b** Von Mises stress of the cross-sections indicated by the dotted lines in **a**. **c** FEA results showing the first principal strain of the surfaces of the two TEGs under a uniaxial strain of 20%. **d** First principal strain of the cross-sections indicated by the dotted lines in **c**. **e** Resistance change as a function of distance between the ends of the TEG and its bending radius. The inset photographs show side views of the bent TEG for different bending radii. Scale bar, 2 cm. **f** Bending cyclic test of the compliant TEG showing stable electrical conductivity during and after bending cycles with bending radius (*r*) ~15 mm. The inset shows an enlarged view of the recorded data. **g**, **h** Experimentally measured TE performance of 36-np-pair-compliant TEG after different bending cycles with different bending directions of *x*-axis (**g**) and *y*-axis (**h**). The each inset is optical image of the bent TEG with the different bending directions of *x*-axis (**g**) and *y*-axis (**h**), respectively. Scale bars, 1 cm. **i** Resistance change as a function of uniaxial strain from 0 to 20%. The inset photographs show the compliant TEG under a strain of 0 and 20%. Scale bar, 1 cm. **j** Stretching cyclic test of the TEG showing stable electrical conductivity during and after stretching cycles with a strain of 10%.

Our TEGs showed high deformability and mechanical reliability under tensile strain compared to those reported in the previous literature because the intrinsically stretchable AgNW electrodes with a low Young’s modulus effectively absorb the applied stress, and the infiltrated PDMS acts as a buffer preventing each leg from being ruptured by extreme deformation. For a systematic analysis, we carried out FEA of the stress and strain distribution at a surface containing interconnects when our TEG with AgNW soft electrodes and a counterpart TEG with Cu plate electrodes were mechanically bent and stretched. When they were bent, extremely high stress occurred across the Cu electrodes compared to that across the AgNW electrodes due to the high Young’s modulus of the Cu plates (~120 GPa), resulting in a high bending stiffness and limited deformability (Fig. 4a, b). When a tensile strain of 20% was applied, the FEA results showed a concentrated strain >250% at the interfaces between the PDMS and the Cu plates, which far exceeds the fracture strain of PDMS, while the AgNW electrodes absorbed the external strain and maintained a maximum strain <150% (Fig. 4c, d). We also experimentally demonstrated the mechanical reliability of our TEG by measuring the resistance change (Δ*R*) to the initial resistance (*R*_0_) and TE performance under different bending and stretching conditions. The $\frac{{{\Delta} R}}{{R_0}}$ of the TEG remained at <50% when the bending radius (*r*) reached ~11 mm (Fig. 4e and Supplementary Fig. 16). The resistance of the TEG was also stable over 1000 bending cycles with a *r* of 15 mm (Fig. 4f). The *V*_OC_ and power at a Δ*T*_Applied_ of 10 K were stably maintained even after 10,000 bending cycles along both *x*-axis (Fig. 4g) and *y*-axis (Fig. 4h and Supplementary Fig. 17). Moreover, our compliant TEG showed stretchability up to 20% with a $\frac{{{\Delta} R}}{{R_0}}$ of 160% (Fig. 4i) and great cyclic reliability under a strain of 10% (Fig. 4j). Especially, while flexible TEGs exploiting nanostructured or thin-film TE materials, e.g. poly(3,4-ethylenedioxythiophene) polystyrene sulfonate (PEDOT:PSS) and carbon nanotubes (CNTs), directly undergo applied mechanical strain and therefore suffer from the strain effect on the Seebeck coefficient^48,49^, the bulk Bi_2_Te_3_ legs used in our compliant TEG are completely strain-free under mechanical deformation (Fig. 4c, d). It is because the intrinsically stretchable electrodes efficiently absorb the mechanical strain as a result of the large Young’s modulus difference between the stretchable interconnects and TE legs. Therefore, the *V*_OC_ of our TEG did not change when the tensile strain was applied (Supplementary Fig. 18). This strain-free effect is still valid when the bending strain is applied to the TEG (Supplementary Fig. 19). There was no noticeable change in *V*_OC_ of our TEG under uniaxial (Supplementary Fig. 20) and biaxial bending conditions (Supplementary Fig. 21). Our TEG also exhibited outstanding long-term humidity tolerance for from 1 to 384 h (16 days), even under harsh temperature and humidity conditions (Supplementary Fig. 22).

### Enhanced TE performance on 3D surfaces via conformability

**Fig. 5:**
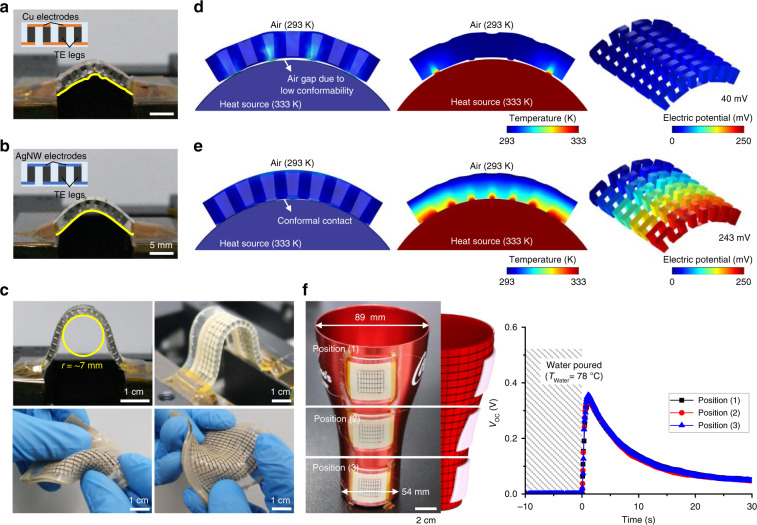
Mechanical conformability of the compliant TEG. **a**, **b** Photographs of bent TEGs comprising Bi_2_Te_3_ legs and Cu electrodes (**a**) and AgNW-based soft electrodes (**b**). The each inset shows schematic illustration of side view of each TEG, respectively. Scale bars, 5 mm. **c** Photographs of the compliant TEGs showing excellent conformability under various deformations. Scale bars, 1 cm. **d**, **e** FEA results showing different deformation and heat transfer behaviors corresponding to TE performance for the TEG with Cu electrodes (**d**) and AgNW-based soft electrodes (**e**). **f** Photographs of a TEG attached to different positions (top, middle, and bottom side) of a bell-shaped aluminum cup. The TEG established conformal contact with the three-dimensional (3D) surface of the aluminum cup. The middle schematic image shows the anisotropic bending curvatures of each position at which the TEG was attached. The right graph shows the time-resolved *V*_OC_ of the TEG attached to the three positions when hot water is poured into the aluminum cup. Scale bar, 2 cm.

Due to this high degree of mechanical freedom and softness of the low-thermal-impedance s-HCs, our TEG could form conformal contact with various 3D heat sources, resulting in significantly enhanced TE performance on them. To clearly show the superior conformability of our compliant TEGs, we fabricated a reference TEG (r-TEG) comprising Bi_2_Te_3_ TE legs and Cu electrodes, which have been widely reported in previous works, and compared its bending deformation with that of our compliant TEG (c-TEG). The r-TEG showed a rugged and angular deformation at the bottom surface and even fractured from the PDMS substrate due to the concentrated tensile strain (Fig. 5a). On the other hand, the c-TEG showed a smooth and free-flowing deformation (Fig. 5b). Moreover, the c-TEG formed a perfect 7-mm-radius circle without fracture (Fig. 5c). To systematically investigate the effect of the improved conformability on TE performance on 3D heat sources, we performed a FEA comparing the *V*_OC_s of the r-TEG and c-TEG that are attached to a curved heat source (Fig. 5d, e). The r-TEG cannot perfectly wrap the curved surface, resulting in undesirable air gaps that substantially impede the heat transfer from the heat source to the bottom surface of the TEG. On the contrary, the c-TEG formed a perfect fit to the curved surface without air gaps, facilitating much better heat transfer to the TE legs. The resulting *V*_OC_ was ~243 mV, ~600% higher than that of the r-TEG. These FEA results highlight that our approach can significantly improve the energy harvesting efficiency of compliant TEGs on 3D heat sources. To further demonstrate reliable energy harvesting on 3D surfaces, we attached the compliant TEG to different positions of a bell-shaped aluminum cup with anisotropic bending curvatures and monitored the *V*_OC_ of the TEG when 78 °C water was poured into the cup (Fig. 5f). The TEG generated a maximum *V*_OC_ of ~340 mV, and no significant difference was observed in the time-resolved *V*_OC_ according to the attachment position, proving the efficient heat collection of our compliant TEG regardless of the shapes of the heat source.

### Self-powered wearable applications

**Fig. 6:**
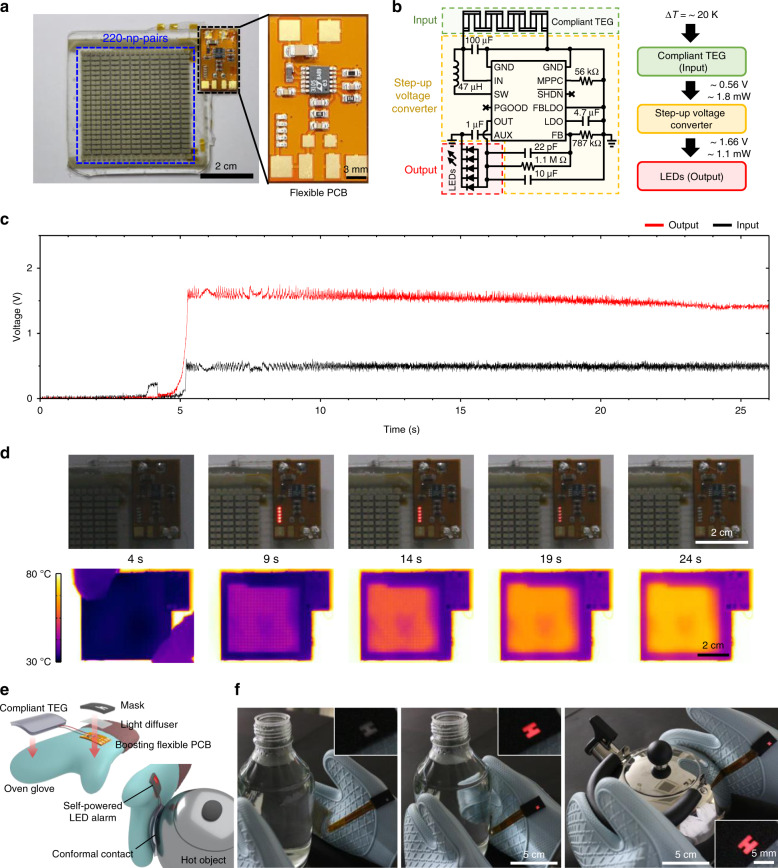
Self-powered wearable applications with a high-performance compliant TEG. **a** Photograph of the large-area compliant TEG and an flexible printed circuit board (f-PCB) with a step-up voltage converter and five light-emitting diodes (LEDs). The right photograph is enlarge view of the f-PCB. Scale bar, 2 cm and 3 mm. **b** Circuit diagram and optical image of the f-PCB for step-up voltage conversion and LED operation. The block diagram shows an operating sequence with the output voltage and power after each block. **c** Input and output voltages of the step-up converter when the TEG was put on a hot plate. **d** Real-time optical and infrared images of the compliant TEG with a step-up voltage converter after the TEG was put on a hot plate. The photographs show that the LEDs was turned on immediately after the TEG was in contact with the hot plate. The infrared images show the temperature of the upper surface of the TEG. Scale bars, 2 cm. **e** Schematic illustration of hot surface warning gloves with a self-powered LED system and light masking packages. **f** Photographs showing a demonstration of the TEG-attached gloves when they are used to grasp various hot objects such as a bottle and a kettle. The insets show enlarged view of the self-powered system and packages. The conformal contact between the TEG-attached gloves and the 3D surfaces of the heat sources results in a bright “H” sign without any assistance from an external power supply. Scale bars, 5, 5 cm, and 5 mm.

To demonstrate a fully self-powered wearable device that provides an alert about an abrupt temperature increase driven by our compliant TEG, we designed a flexible printed circuit board (f-PCB) with a step-up voltage converter and five light-emitting diodes (LEDs) and integrated it with the 220-np-pair TEG (Fig. 6a, b and Supplementary Fig. 23 for details). When Δ*T*_Applied_ was ~20 K, the TEG generated ~1.8 mW at 0.56 V, and the output voltage of the step-up converter was ~1.66 V with ~1.1 mW, which was sufficient to turn on the LEDs. Notably, our flexible circuit system was only powered by the compliant TEG without additional power supply. The minimum Δ*T*_Applied_ required to turn on the LEDs was calculated to be ~13 K (Supplementary Fig. 24). Figure 6c shows the input and output voltages of the step-up converter and the resulting LED operation when the TEG was placed on a hot plate for a sufficient Δ*T*_Applied_. The input and output voltages increased to ~0.56 and ~1.66 V, respectively, instantaneously turning on the LEDs after the TEG was placed on the hot plate, and then, the generated voltages gradually decreased as the thermal equilibrium state was reached (Fig. 6d). We further demonstrated “hot surface warning gloves” by integrating the self-powered LED system and light masking packages into oven gloves (Fig. 6e). When the TEG-attached gloves were used to grasp various hot objects, such as a glass bottle and a kettle, the conformal contact between our TEG and the 3D surfaces resulted in a bright “H” sign due to the LEDs being turned on, without the assistance of external power (Fig. 6f and Supplementary Movie 2). This demonstration highlights the feasibility of our high-performance compliant TEG in practical wearable applications.

## Discussion

We introduced a highly conformable TEG with high TE performance arising from an improved heat transfer ability. Magnetically self-assembled Ag–Ni particles in an elastomer significantly enhance the heat transfer to the TE legs, while maintaining its softness, exhibiting a thermal conductivity of ~1.4 W m^−1^ K^−1^ and Young’s modulus of <10 MPa. The s-HCs maximize the $\frac{{{\Delta} T_{{\mathrm{TE}}}}}{{{\Delta} T_{{\mathrm{Applied}}}}}$ up to ~80%, resulting in a ~260% improvement in the output power compared to the TEG without s-HCs. The FEA results further support the drastic enhancement in heat transfer ability via s-HCs and consequent improvement in TE performance. In addition, intrinsically stretchable interconnects effectively absorb strain energy, improving mechanical reliability and deformability of our TEG that is even stretchable up to ~20%. The approach facilitates highly conformal contact with arbitrary 3D surfaces, thus significantly minimizing energy loss in undesirable air gaps, which has been a chronic limitation of previous flexible and wearable TEGs using rigid interconnects. Due to their excellent conformability, our TEG achieves the highest TE performance on human skin and operates a self-powered wearable warning system for arbitrary hot features, proving its superiority in energy harvesting on 3D heat sources. Importantly, our automated additive manufacturing with high device yield under ambient conditions realizes low cost, high FF, and highly customizable TEGs beyond laboratory-scale applications, shedding light on mass production of wearable TEGs. Future work would include further improvement in TE performance by reducing the electrical resistance of the stretchable interconnects and the contact resistance between the adhesive materials and electrodes. This direction, together with our approaches for realizing highly compliant TEGs, could significantly minimize waste heat from changing 3D heat sources, paving the way toward the realization of wearable and self-powered IoT devices.

## Methods

### Materials

Trichloro(1*H*,1*H*,2*H*,2*H*-perfluorooctyl)silane (FOTS) (Sigma Aldrich), PDMS (Dow Corning), AgNW solution (10 g L^−1^, dispersed in ethanol) (Yurui Chemical Co., Ltd), silver-coated nickel (Ag–Ni) particles (SN08P40, Potters), conductive silver epoxy (ABLEMOND 84-1LMISR4, Ablestik), and the TE material bismuth telluride (Bi_2_Te_3_) (Kyrotherm) were used as received.

### Soft heat transfer and electrical interconnection platform

Before depositing AgNW electrodes, surface treatment with vapor-deposited FOTS was conducted on a 125-μm-thick PEN carrier substrate to ensure sufficient hydrophobic properties that facilitate detachment of the AgNW electrodes. On the prepared PEN substrate, which was on a 70 °C hot plate, 2 mL of a AgNW solution was spray-coated using a patterned metal mask and a portable airbrush (DH-125, Sparmax). Ag–Ni particles were mixed in PDMS and curing agent with 10:1 in a weight ratio using a paste mixer (ARE-310, Thinky). The Ag–Ni particles/PDMS precursor mixture was poured onto an FOTS-treated 400-μm-thick glass substrate with 120-μm-thick spacers. The prepared AgNW-coated PEN was placed on top of the mixture. The mixture covered by the PEN substrate was sandwiched by two iron pillar arrays (1.2 × 1.2 mm^2^ pillar, 9 × 9 arrays) and a designed mold, and then, two vertically aligned magnets were attached at the top and bottom of the pillar arrays. The magnetic flux intensity of two magnets was ~1600G for all s-HCs fabricated in this work, if not specified. Because the magnetic field was applied from before the curing process, the Ag–Ni particles could freely travel in the uncured PDMS precursor matrix. The randomly distributed Ag–Ni particles in the mixture rapidly converged into the location of the iron pillars and were vertically aligned in the direction of the applied magnetic field. This behavior was completed as soon as the magnetic field was applied (<~180 s), and then the Ag–Ni particles did not move anymore. After sufficient resting (~10 min), we cured the static mixture in a 100 °C oven for 1 h, maintaining the magnetic field until the curing process was completed. After curing the mixture and detaching the PEN substrate, a SHEP on the supporting glass was produced.

### Fully automated processes for transferring Bi_2_Te_3_ TE legs

The highly conductive epoxy was directly printed in the areas where the s-HCs with AgNW electrodes were located using a programmable pneumatic dispenser (SHOTmini 200Sx, Musashi Eng.) and a 32G needle with a pressure of 400 kPa for 2 s. The epoxy layer was used as an adhesive layer that delivered a lower contact resistance between the Bi_2_Te_3_ TE legs and AgNW electrodes. Then, the Bi_2_Te_3_ legs were sequentially placed by a programmable pick-and-place machine (TM220A, NeoDen) on the printed epoxy. After annealing the bottom layer at 170 °C for 1 h in a oven, the other s-HC platform was attached to the top of the Bi_2_Te_3_ legs and annealed under the same conditions. For the realization of mechanically reliable and compliant TEGs, PDMS infiltration between the top and bottom platforms was performed, followed by annealing at 100 °C for 1 h on a hot plate. Finally, compliant TEGs with s-HCs and intrinsically stretchable electrodes were realized after the supporting glasses were detached from the TEGs.

### TE characterization of compliant TEGs

To ensure a controlled temperature difference between the Bi_2_Te_3_ TE legs, Peltier devices and air coolers were used for heating and cooling, respectively. The compliant TEGs were sandwiched between the heated and cooled metal plates to measure their TE characteristics. During the measurement, nonconductive thermal pads were attached onto the top and bottom of the TEGs to prevent electrical shorting among adjacent heat conductors. The temperature difference between the hot and cool zones was monitored via a thermometer with a thermocouple wire placed between the thermal pad and the TEGs. After the thermal equilibrium state was reached, the output voltage and current were measured by a SourceMeter (Keithley 2400). The Seebeck coefficients of p- and n-type Bi_2_Te_3_ legs were calculated by measuring the Seebeck voltage of each leg when the temperature difference was −10, −5, 5, and 10 K, respectively. The Bi_2_Te_3_ leg is sandwiched by highly conductive thin aluminum (Al) films (16 μm thickness) to minimize the vertical parasitic heat loss and the temperature difference across the leg was controlled by two Peltier devices (Supplementary Fig. 25 for details). The thermal conductivity was characterized from the measurements of the density, specific heat, and thermal diffusivity of the bulk heat conductors. The density was measured according to the standard test methods for density and specific gravity of plastics by displacement (ASTM D792) using an electronic densimeter (MD-300S, Alfa Mirage). The specific heat was measured according to Plastics-differential scanning calorimetry (DSC)—Part 4: Determination of specific heat capacity (ISO 11357-4) using DSC equipment (DSC 25, TA). The thermal diffusivity was measured according to the standard test method for thermal diffusivity by the flash method (ASTM E1461) using a thermal diffusivity measurement system (NETZSCH, LFA 447 NanoFlash).

### Design of flexible circuits and self-powered wearable warning system

To turn on the LEDs to act as a warning sign, a step-up voltage converter was used to step up the generated voltage from the compliant TEG. The step-up voltage converter was implemented on an f-PCB to maintain conformal contact with our compliant TEG and was composed of a voltage regulator (LTC3105, Linear Technology), capacitors (22 pF, 1 μF, and 10 μF, 0603 package, and 100 μF, 1206 package), resistors (56.7 kΩ, 787 kΩ, and 1.1 MΩ, 0603 package), an inductor (47 μH, 0603 package), and red LEDs (SML-P11UT, 0402 package). These passive components were optimized considering the specifications of the loaded LEDs with a turn-on voltage of ~1.6 V. Based on the low start-up voltage (250 mV) and maximum power point controller, the system was designed to be operated by only the generated voltage from our compliant TEGs without the assistance of any additional power supply. To provide the warning text, H, a thin light diffuser to diffuse light from five LEDs was placed over the designed step-up voltage converter system. The self-powered wearable warning system was attached to oven gloves, which were used to hold a bottle and a kettle containing hot water.

### Optical, infrared, SEM, and EDS observations

Surface and cross-section images of the s-HCs with AgNW interconnects were captured using optical microscopy (DSX510, Olympus). The real-time heat transfer of our s-HCs was examined using an infrared thermal imaging camera (T420, FLIR Systems). The distribution images of Ag–Ni particles in the PDMS layers with and without magnetic self-assembly were captured using SEM (Sigma 300, ZEISS) and analyzed by EDS (XFlash6160, Bruker).

### Mechanical reliability of the s-HCs and the compliant TEG

Strain–stress curves of our s-HCs with different Ag–Ni weight ratios (50, 60, and 70 wt%) were compared to those of a bare PDMS layer and commercially available thermal pads (H48-2K, H48-6G, and TGX, t-Global Technology). Tensile strain tests were conducted using a universal testing system (UTM, Instron 5567, Instron) with a strain rate of 20 mm min^−1^. The width, length, and thickness of the s-HCs were 10, 20, and 0.1 mm, respectively. In case of commercial thermal pads, the width and length of the counterparts were the same as those of our heat conductors, but the thicknesses were different (H48-2K, H48-6G, and TGX: 0.1, 0.3, and 0.5 mm thicknesses, respectively).

To investigate the mechanical reliability of the compliant TEG, the resistance and TE performance of our compliant TEG were measured during and after different bending and stretching conditions. Using 1D tensile equipment and a SourceMeter (Keithely 2400), a cyclic bending and stretching test to confirm resistance change of the TEG were conducted with a bending radius of 15 mm at a rate of 100 mm min^−1^ for bending and a strain of 10% at a rate of 20 mm min^−1^ for stretching, respectively. The TE performance was measured after bending cycles along both *x*-axis and *y*-axis (Supplementary Fig. 17). The *V*_OC_ under the applied strain from 0 to 7% was also measured (Supplementary Fig. 18).

To confirm humidity and temperature tolerance, the TE performance of our compliant TEG was measured after exposing it to different humidity and temperature environments for from 1 to 384 h. The humidity and temperature are controlled by a humidifier and hot plate, respectively, in a glass box, where the humidity is measured by a hygrometer in real time. The *V*_OC_ and maximum power were measured at a given temperature difference of 10 K after exposing the TEG for 1 h in the different humidity (30 and 80%) and temperature (293, 323, and 353 K) conditions. Acceleration tests were also conducted to verify the effect of exposure time to high humidity on the TE performance while maintaining the humidity at 80% and temperature at 323 K for 384 h (Supplementary Fig. 22).

### 3D FEA for calculating the performances of the compliant TEG

The heat transfer in solids, electric currents, and solid mechanics modules in COMSOL Multiphysics (COMSOL Inc.) were used to estimate the TE and mechanical performances of our compliant TEG. To calculate the TE effect of the compliant TEG, the thermal conductivity of the s-HC was extracted from the extrapolation of the measured data, and the electrical conductivity of the TE leg from the measured data was used. Except as stated above, the properties of materials were taken from the datasheet, or the given default values in COMSOL were used. The dimensions of all compliant TEGs for modeling were the same as those of the real device. The performances of 36-np-pair-compliant TEGs without and with s-HCs were characterized by the TE effect module using multiphysics simulations. A comparison of the heat transfer abilities of compliant TEG units without and with s-HCs was carried out using the heat transfer in solids module. To show the high deformability and mechanical reliability of our compliant TEG, bending and stretching simulations using the solid mechanics module were conducted according to the type of electrode, such as Cu plates and embedded AgNW electrodes.

## Supplementary information

Supplementary Information

Description of Additional Supplementary Files

Supplementary Movie 1

Supplementary Movie 2

## Data Availability

The authors declare that all data supporting the findings of this study are available within the article and its Supplementary Information files or from the corresponding author upon reasonable request.
