# Author Correction: High-performance compliant thermoelectric generators with magnetically self-assembled soft heat conductors for self-powered wearable electronics

**DOI:** 10.1038/s41467-021-21629-y

**Published:** 2021-02-19

**Authors:** Byeongmoon Lee, Hyeon Cho, Kyung Tae Park, Jin-Sang Kim, Min Park, Heesuk Kim, Yongtaek Hong, Seungjun Chung

**Affiliations:** 1 Department of Electrical and Computer Engineering, Inter-University Semiconductor Research Center (ISRC), Seoul National University, Seoul, 08826 Korea; 2 Soft Hybrid Materials Research Center, Korea Institute of Science and Technology, Seoul, 02792 Korea; 3 Institute of Advanced Composite Materials, Korea Institute of Science and Technology, Wanju, Jeonbuk 55324 Korea; 4 KHU-KIST Department of Converging Science and Technology, Kyung Hee University, Seoul, 02447 Korea; 5 Present Address: Soft Hybrid Materials Research Center, Korea Institute of Science and Technology, Seoul, 02792 Korea

**Keywords:** Devices for energy harvesting, Thermoelectric devices and materials, Electrical and electronic engineering

Correction to: *Nature Communications* 10.1038/s41467-020-19756-z, published online 23 November 2020.

Affiliation 1 incorrectly read ‘Department of Electronic and Computer Engineering, Inter-University Semiconductor Research Center (ISRC), Seoul National University, Seoul, 08826, Korea’ instead of the correct ‘Department of Electrical and Computer Engineering, Inter-University Semiconductor Research Center (ISRC), Seoul National University, Seoul, 08826, Korea’.

