# Supplementary material for: High-performance compliant thermoelectric generators with magnetically self-assembled soft heat conductors for self-powered wearable electronics
Source: Nat Commun. 2020 Nov 23;11:5948. doi: 10.1038/s41467-020-19756-z (PMC7684283; doi:10.1038/s41467-020-19756-z)
Supplement: Supplementary file 1 — Supplementary Information [file 41467_2020_19756_MOESM1_ESM.pdf]

Supplementary Information

High-performance compliant thermoelectric generators with magnetically self-assembled soft heat conductors

Lee *et al.*

Supplementary Figures

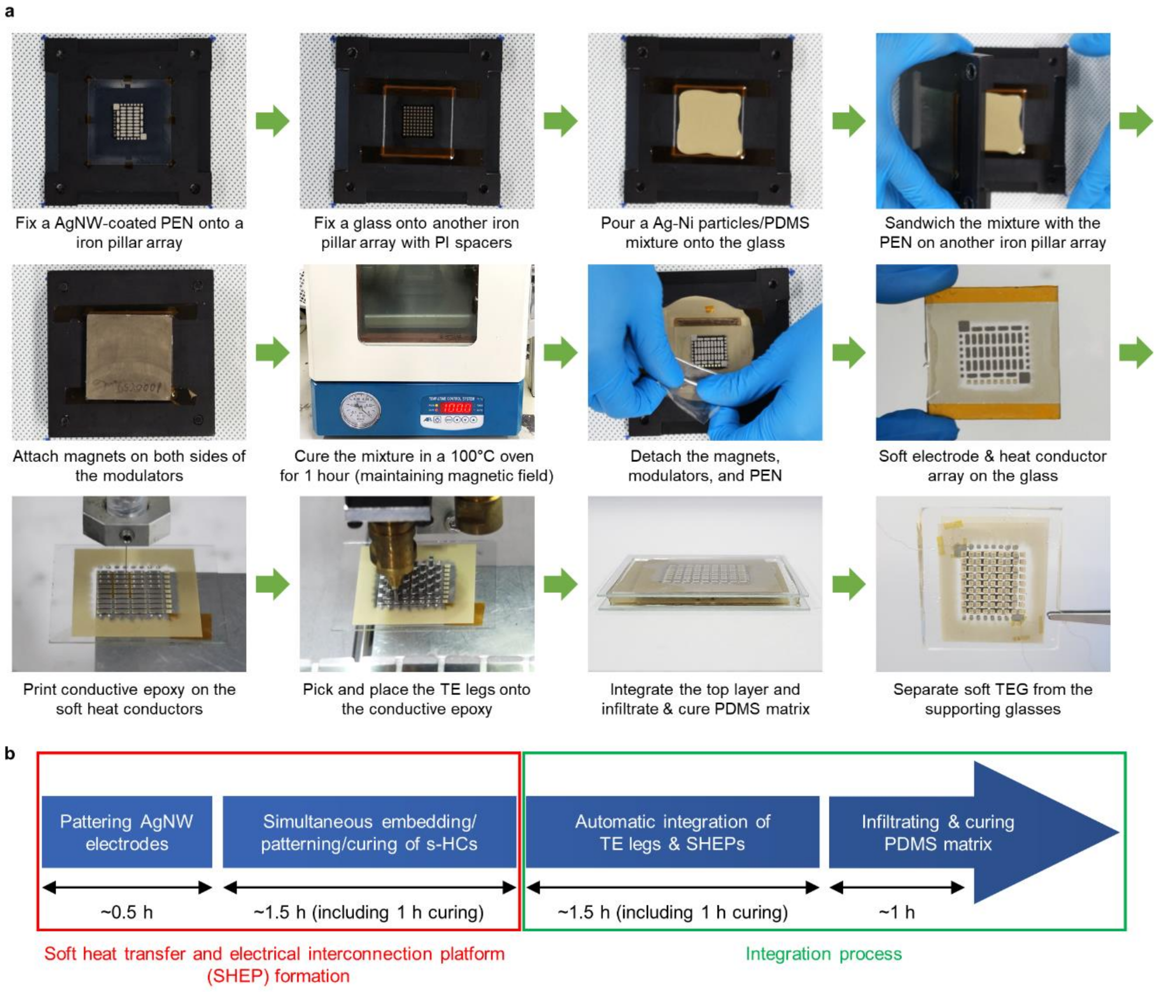

Supplementary Figure 1 | Fabrication process for the compliant thermoelectric generator (TEG). a, Detailed procedures for the fabrication of the compliant TEG with optical images. **b**, Process time for each fabrication step.

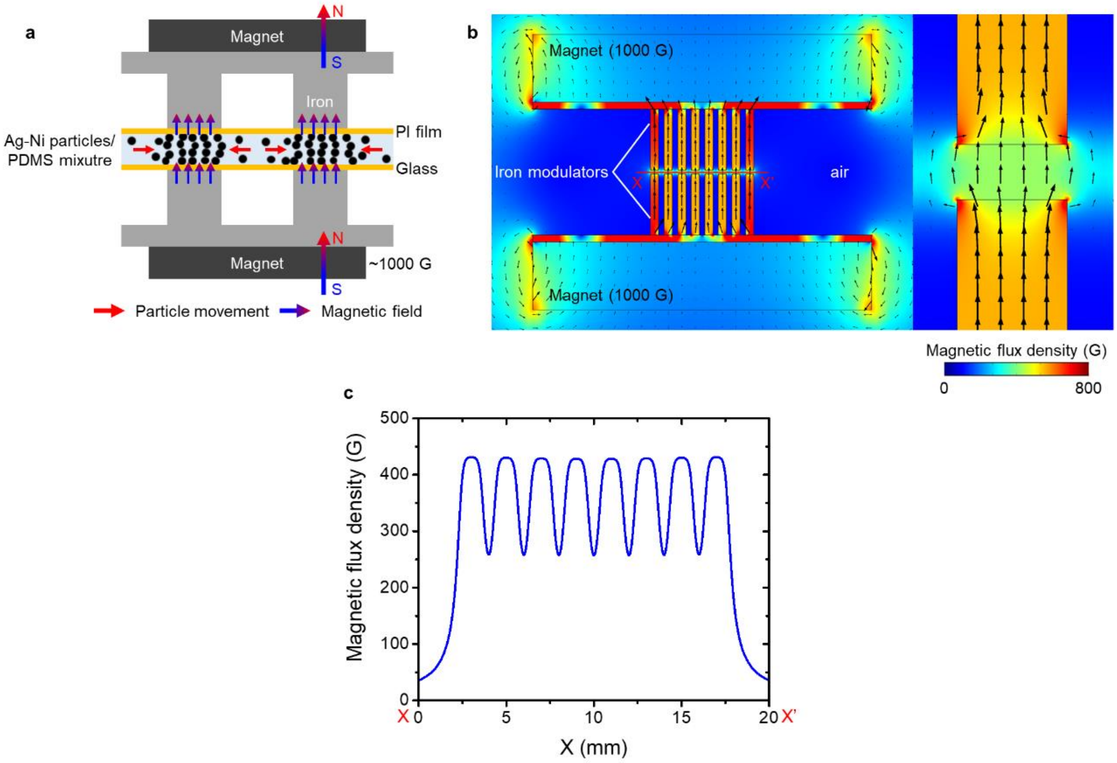

Supplementary Figure 2 | Magnetic self-assembly process for soft heat conductors (s-HCs). a, Schematic illustration for the movement of silver-coated nickel (Ag-Ni) particles in the polydimethylsiloxane (PDMS) precursor under the magnetic field applied through the iron pillar arrays. **b**, Finite element analysis (FEA) results showing the magnetic flux intensity when the magnets were attached to the both sides of the two iron pillar arrays. The actual dimensions of the TEGs were used in the simulation. **c**, Line profile of magnetic flux intensity at the line xx' in **b**.

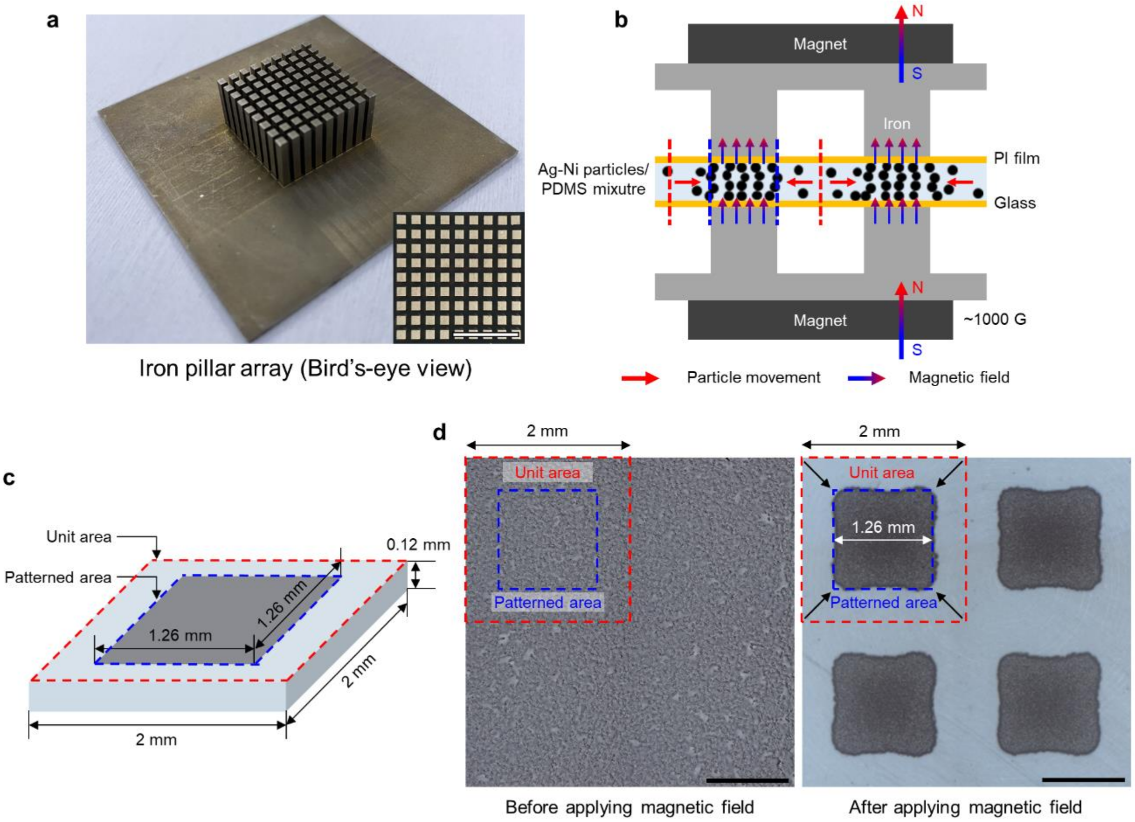

Supplementary Figure 3 | Ag-Ni particle distribution before and after the magnetic self-assembly process. a, Optical image of the iron pillar array as a magnetic field modulator. The inset is a top-view optical image of the iron pillar array. Scale bar, 1 cm. **b**, Schematic illustration for the Ag-Ni particle movement in the PDMS precursor under the magnetic field applied through the iron pillar arrays. **c**, Schematic illustration defining a unit area and patterned area for each s-HC pattern. **d**, Optical images showing Ag-Ni particle distribution before and after the magnetic self-assembly process. Scale bars, 1 mm.

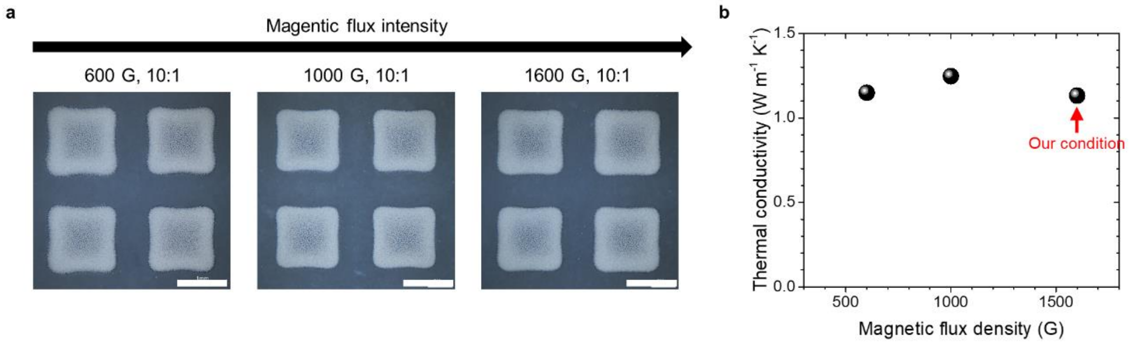

Supplementary Figure 4 | s-HCs fabricated under different magnetic flux intensities. a, Top-view optical images of the s-HC patterns using magnets with 600 G, 1000 G, and 1600 G. Scale bars, 1 mm. **b**, Thermal conductivities of the s-HCs as a function of magnetic flux density.

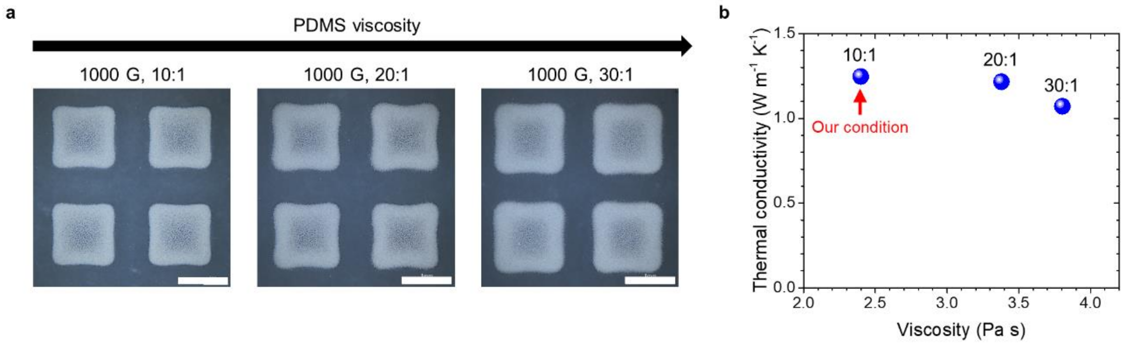

Supplementary Figure 5 | s-HCs patterns with different PDMS viscosities. a, Top-view optical images of the s-HC patterns with a PDMS mixing ratio of 10:1, 20:1, and 30:1. Scale bars, 1 mm. **b**, Thermal conductivities of the s-HCs as a function of viscosity of the PDMS precursor mixture.

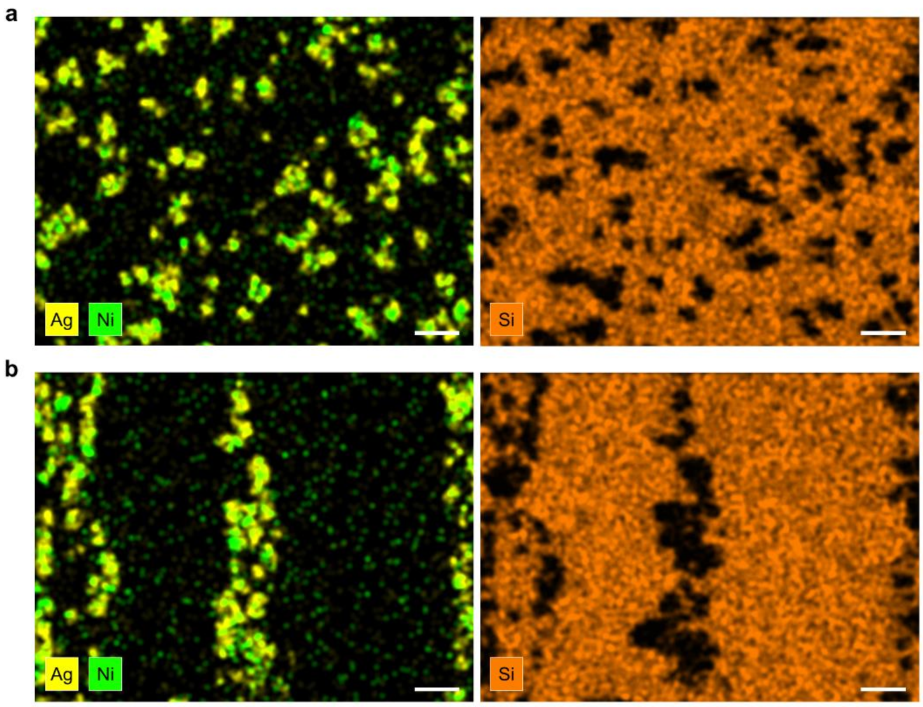

Supplementary Figure 6 | Ag-Ni particles distribution in elastomer matrix according to magnetic self-assembly. a and **b**, Energy dispersive spectrometry (EDS) images of bulk Ag-Ni particle/PDMS composites showing Ag, Ni, and Si distribution without (**a**) and with magnetic self-assembly (**b**). Scale bars, 40 µm.

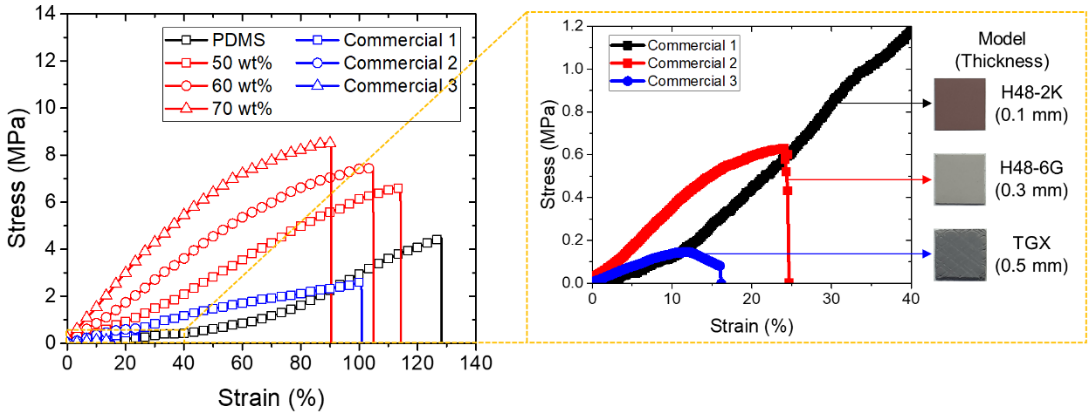

Supplementary Figure 7 | Comparison of strain-stress curves with commercial products. Strain-stress curves of a bare PDMS and magnetically self-assembled Ag-Ni particle/PDMS composites with different Ag-Ni particle concentration (50, 60, and 70 wt%) and three commercial thermal pads. The right plot is an enlarged view of strain-stress curves of three commercial thermal pads with a different thickness.

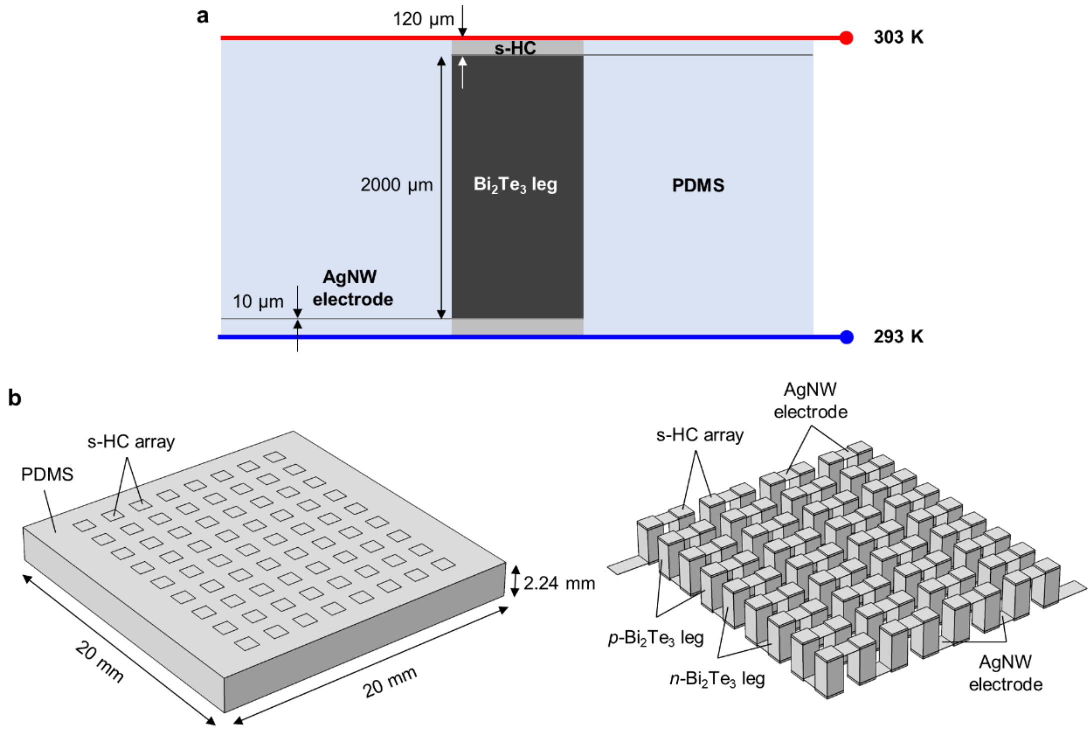

Supplementary Figure 8 | Three-dimensional (3D) FEA modeling for calculating thermoelectric (TE) performance of compliant TEGs. a, Schematic illustration of unit compliant TEG with a single bismuth telluride (Bi_2Te_3) leg for 3D FEA modeling. The TEGs without and with s-HC were characterized for a given temperature difference of 10 K. **b**, Modelling details for the 3D FEA simulation. The left schematic illustration shows dimensions of 36-np-pair TEG with s-HC array and PDMS medium. The right illustration shows inside view of the TEG with hiding the PDMS to indicate each component, such as s-HC, silver nanowire (AgNW) electrodes, and Bi_2Te_3 leg.

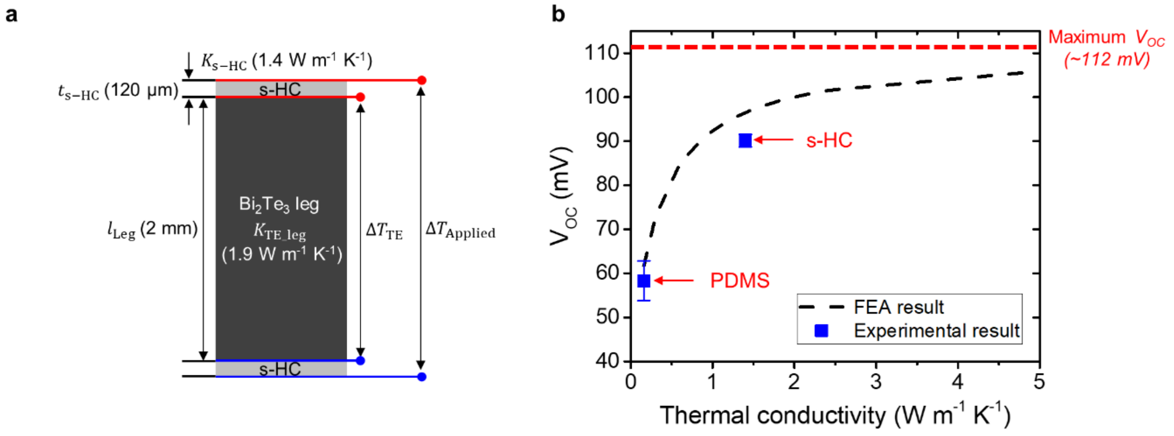

Supplementary Figure 9 | Effect of thermal conductivity of s-HC on open-circuit voltage. **a**, Schematic illustration of a single TE leg with s-HC. **b**, Theoretically calculated and experimentally measured open-circuit voltage (V_{OC}) of a 36-np-pair TEG as a function of thermal conductivity of the s-HC. The maximum V_{OC} was calculated by considering Seebeck coefficient of the TE leg for a given temperature difference of 10 K.

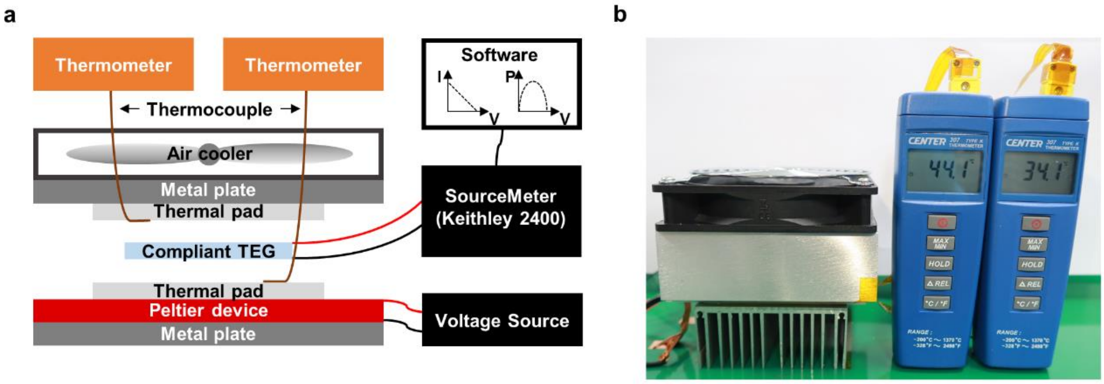

Supplementary Figure 10 | TE characterization of compliant TEG. a and **b**, Schematic illustration (**a**) and photograph (**b**) of a homemade measurement equipment for introducing temperature differences and measuring the TE performance of the fabricated 36-and 220-np-pair compliant TEGs.

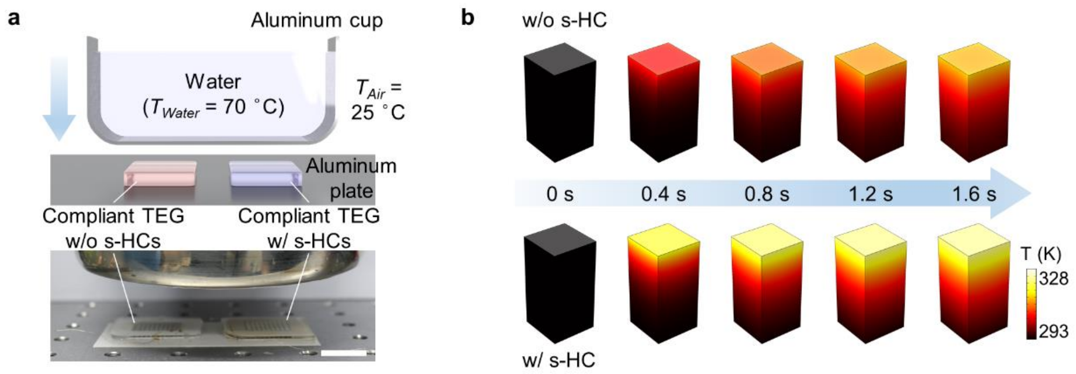

Supplementary Figure 11 | Dynamic response of the compliant TEG. a, Schematic illustration and photograph of the experimental setup for measuring the response of the TEGs without and with s-HCs when an aluminum cup with hot water is abruptly placed in contact with two TEGs. Scale bar, 2 cm. **b**, FEA results visualizing the temperature distribution in a single TE leg. The TEG with s-HCs shows faster heat transfer from the heat source and large temperature gradient.

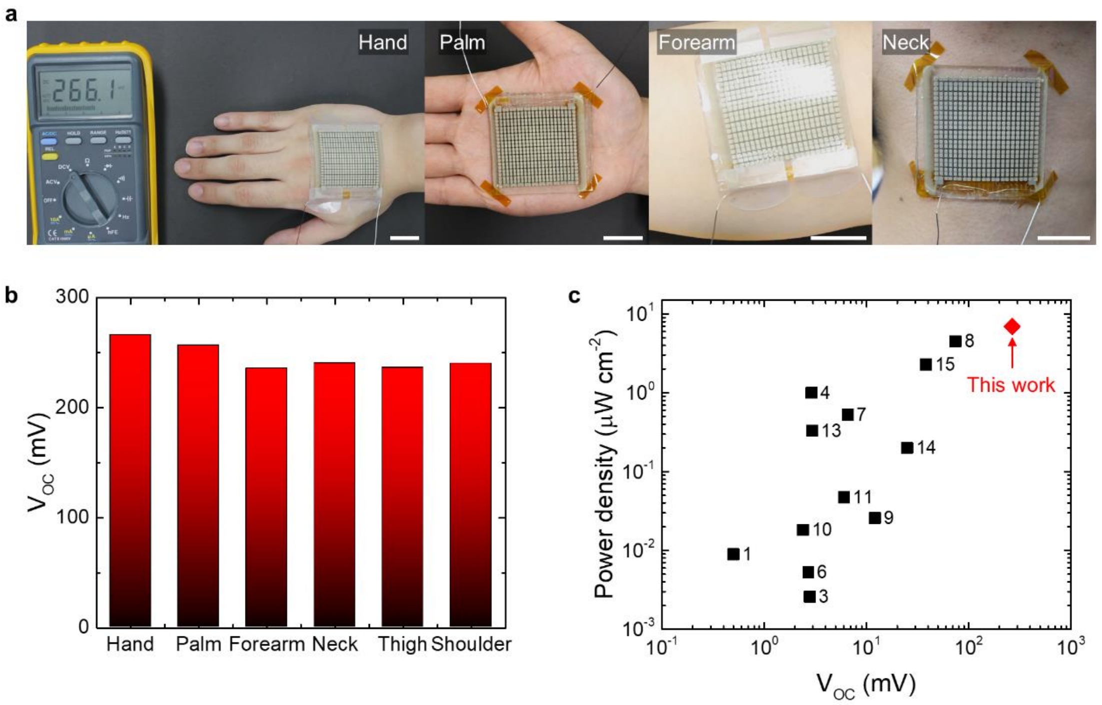

Supplementary Figure 12 | TE performance of the compliant TEG on human skin. a, Photograph of generated V_{OC} from a compliant TEG on human hand. The right three photographs show forming conformal contacts on various human skins (palm, forearm, and neck). Scale bars, 2 cm. **b**, Experimentally measured V_{OC} of the compliant TEG showing superior TE performance on various human skins (hand, palm, forearm, neck, thigh, and shoulder). **c**, Comparison of power density and V_{OC} of wearable TEGs attached to human skin without heat sinks.

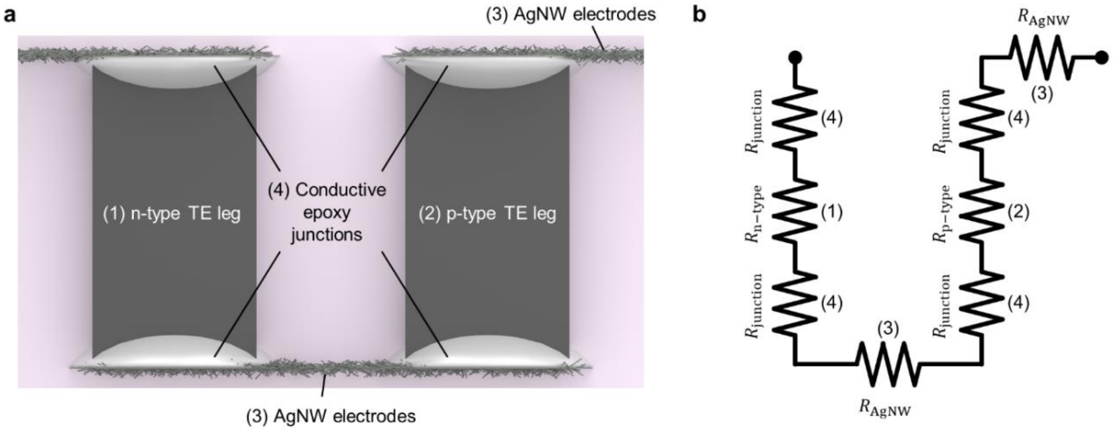

Supplementary Figure 13 | Resistor model of the compliant TEG. a, Resistive components in the compliant TEG. A unit component set includes an n-type TE leg, p-type TE leg, two AgNW-based stretchable electrodes, and four conductive epoxy junctions. **b**, Equivalent resistor model for the unit component set in **a**.

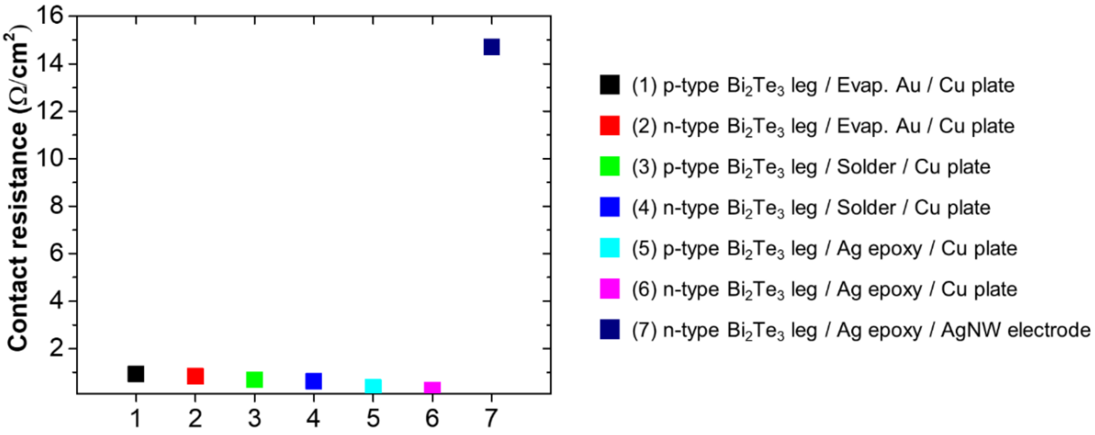

Supplementary Figure 14 | Measured resistance of various junction configurations.

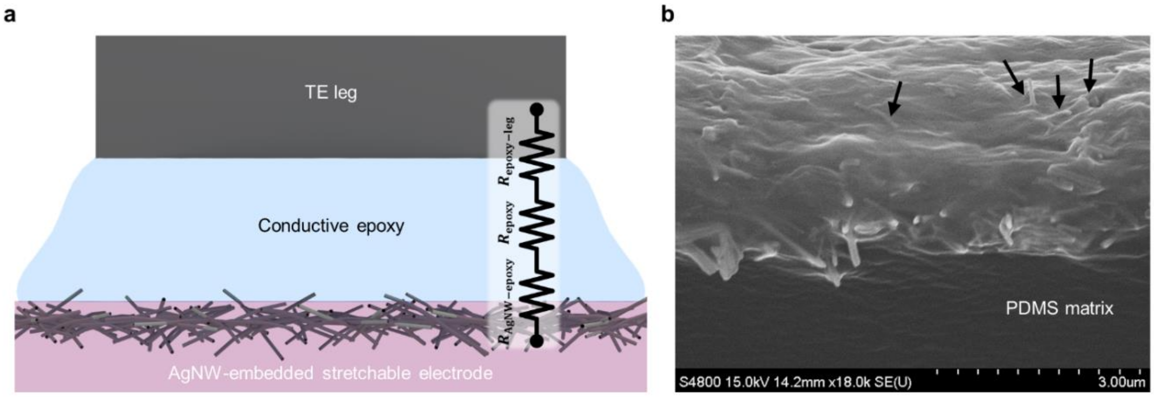

Supplementary Figure 15 | Conductive epoxy junction between TE leg and AgNW stretchable electrode. a, Schematic illustration of conductive epoxy junction between TE leg and AgNW-based stretchable electrode, and its resistor model. **b**, Scanning electron microscopy (SEM) image showing the surface of the AgNW-based stretchable electrode. Black arrows indicate the AgNWs exposed on a surface of the PDMS matrix.

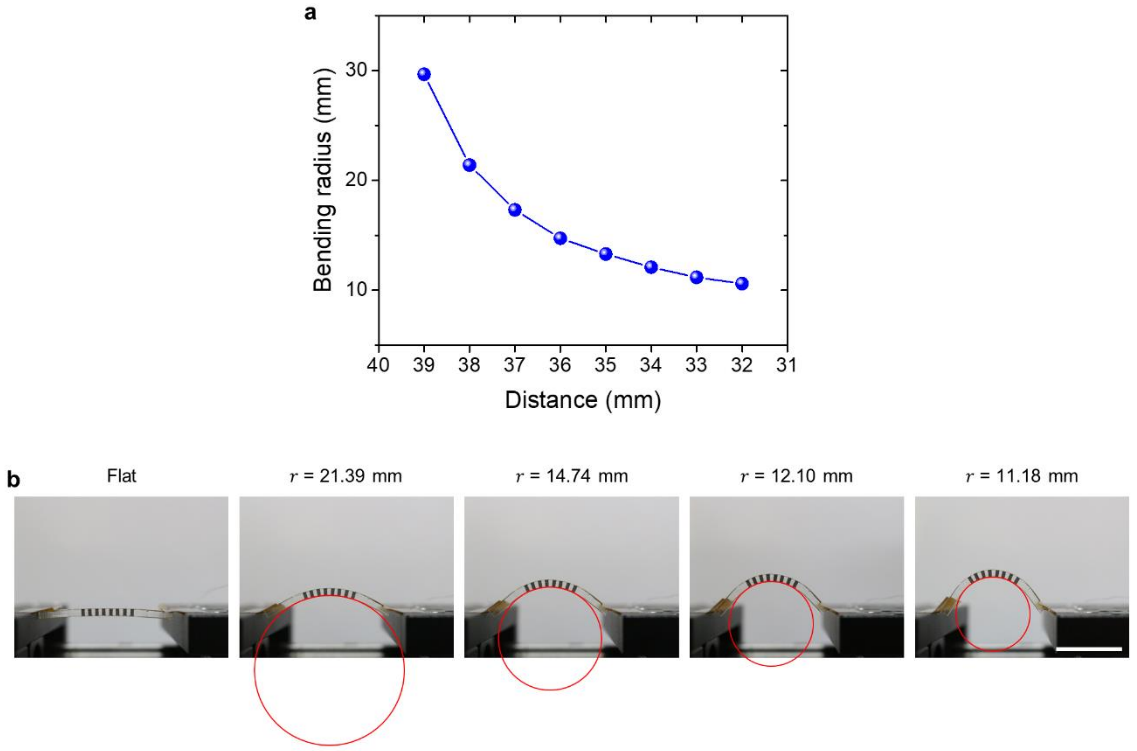

Supplementary Figure 16 | Bending radius of compliant TEG. a, Bending radius (*r*) change as a function of distance between both ends of compliant TEG. The distance is decreased from 40 to 32 mm. **b**, Photographs of side-view of bent compliant TEG for different bending radii, Scale bar, 2 cm.

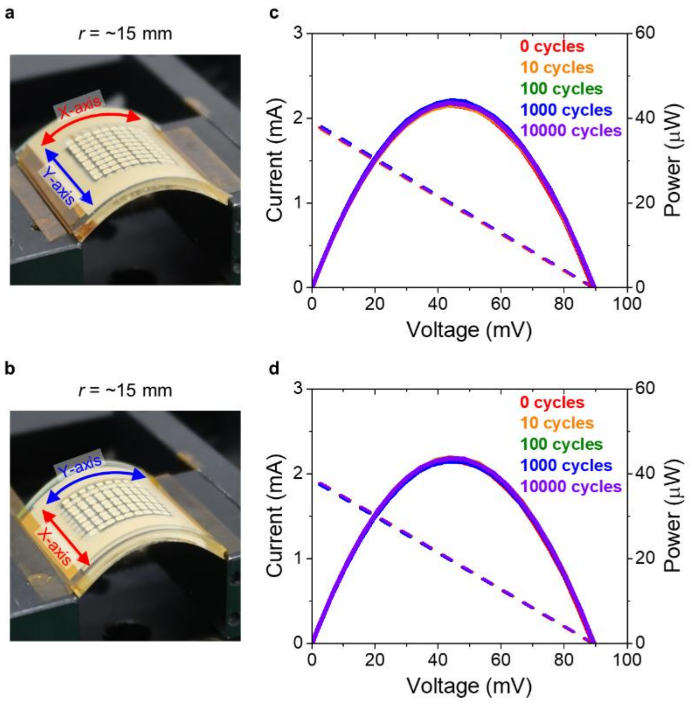

Supplementary Figure 17 | TE performance of the compliant TEG under different bending conditions. a and **b**, Optical images of the bent TEGs with the different bending directions of x-axis (**a**) and y-axis (**b**). **c** and **d**, Experimentally measured TE performance of 36-np-pair TEGs after different bending cycles with different bending directions of x-axis (**c**) and y-axis (**d**).

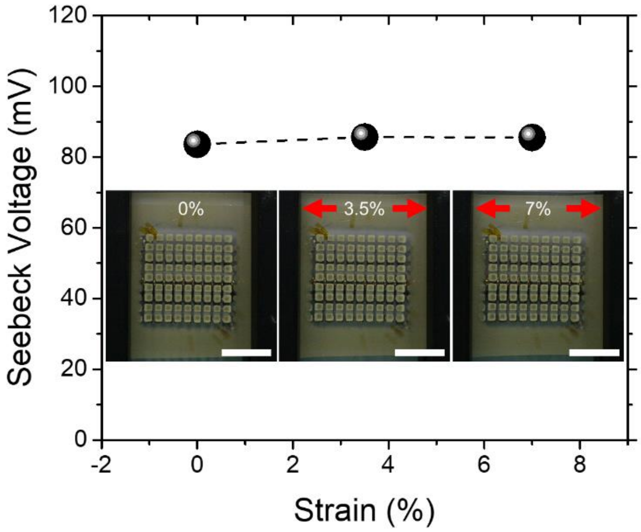

Supplementary Figure 18 | TE performance of the compliant TEG under tensile strain. Experimentally measured Seebeck voltages generated by 36-np-pair TEG at a temperature difference of 10 K when different strains applied. Scale bar, 1 cm.

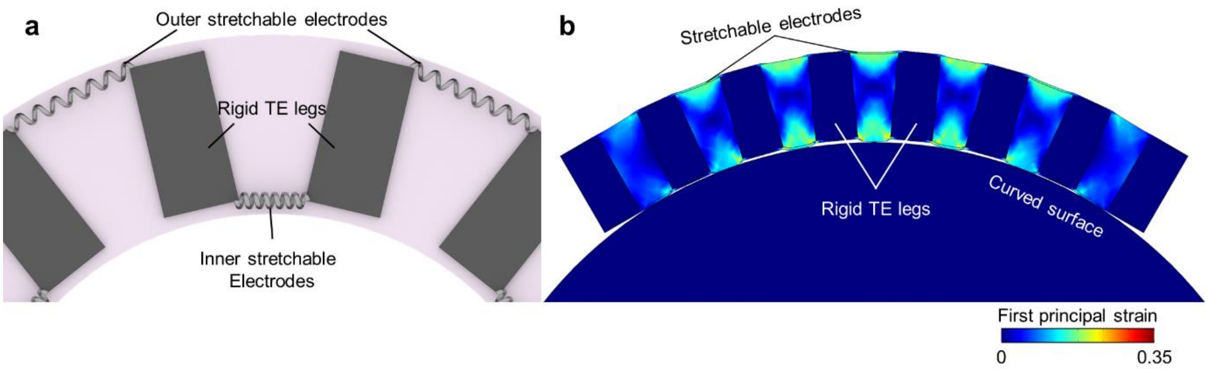

Supplementary Figure 19 | Strain-free effect under bending conditions. a, Schematic illustration of strain-free effect of rigid TE legs while bending strain is applied. Intrinsically stretchable electrodes are depicted as springs. **b**, FEA result showing the first principal strain of the cross-section of the compliant TEG while bending strain is applied.

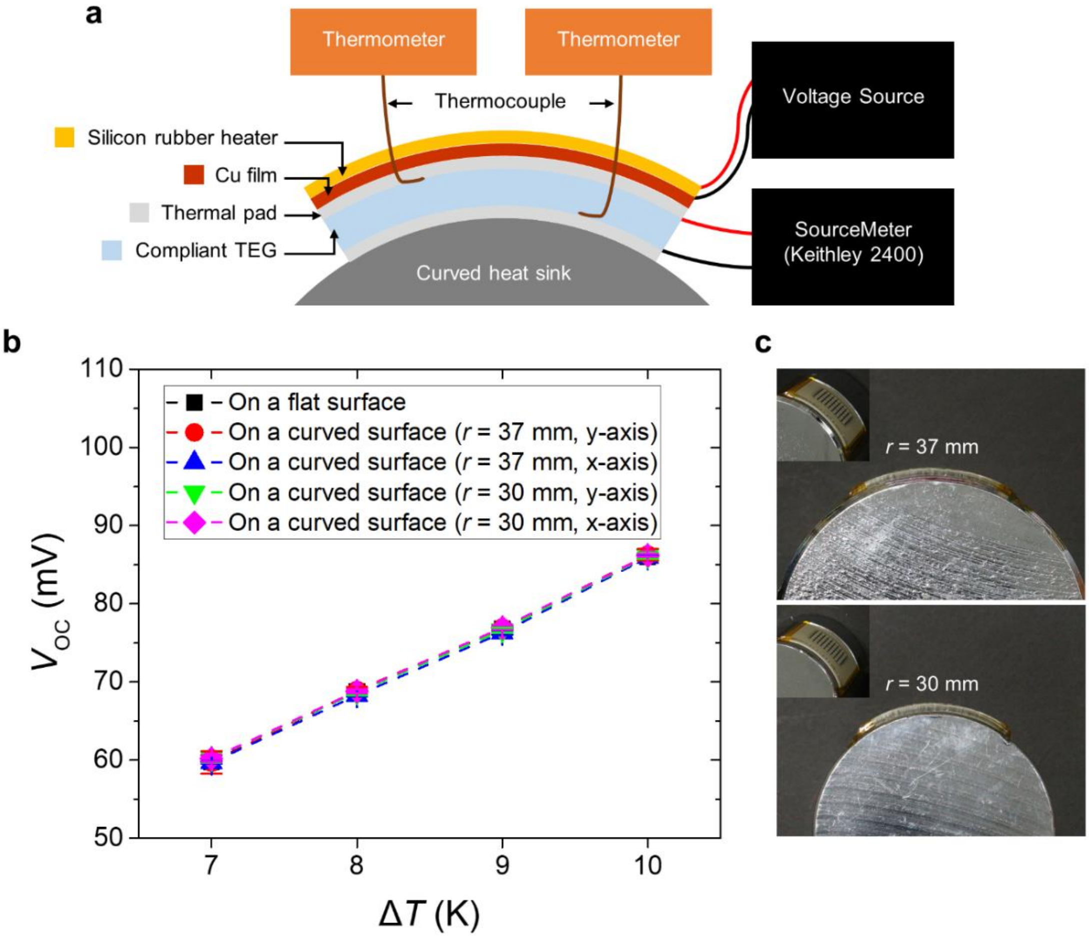

Supplementary Figure 20 | Seebeck coefficient under bending conditions. a, Schematic illustration of the experimental setup for Seebeck voltage measurement under bending conditions. **b**, Seebeck voltage generated by the 36-np-pair TEG at temperature difference (ΔT) values of 7, 8, 9, and 10 K under different bending conditions. The error bars represent the standard deviation. **c**, Photographs of the TEG attached to curved heat sinks with bending radii of 37 mm and 30 mm, respectively.

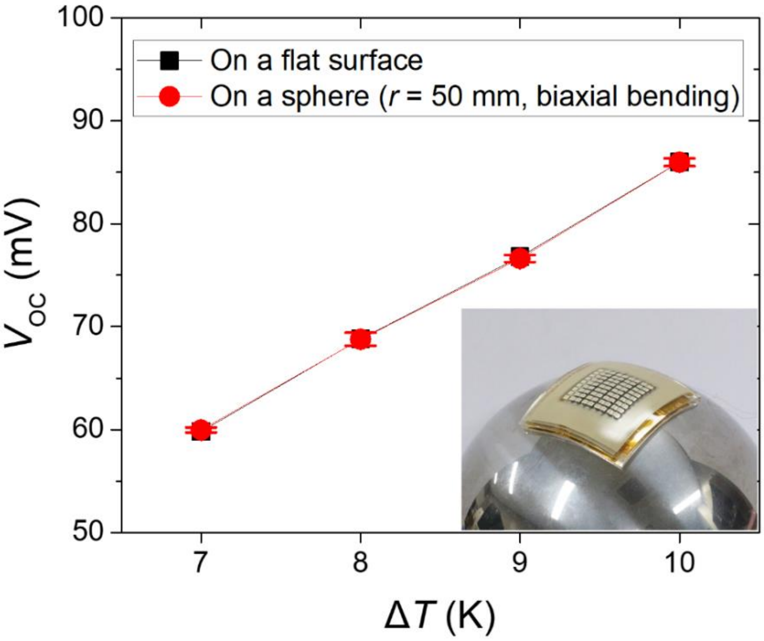

Supplementary Figure 21 | Seebeck coefficient under a biaxial bending condition. The inset is the photograph of the TEG attached to a spherical heat sinks with a radius of 50 mm. The error bars represent the standard deviation.

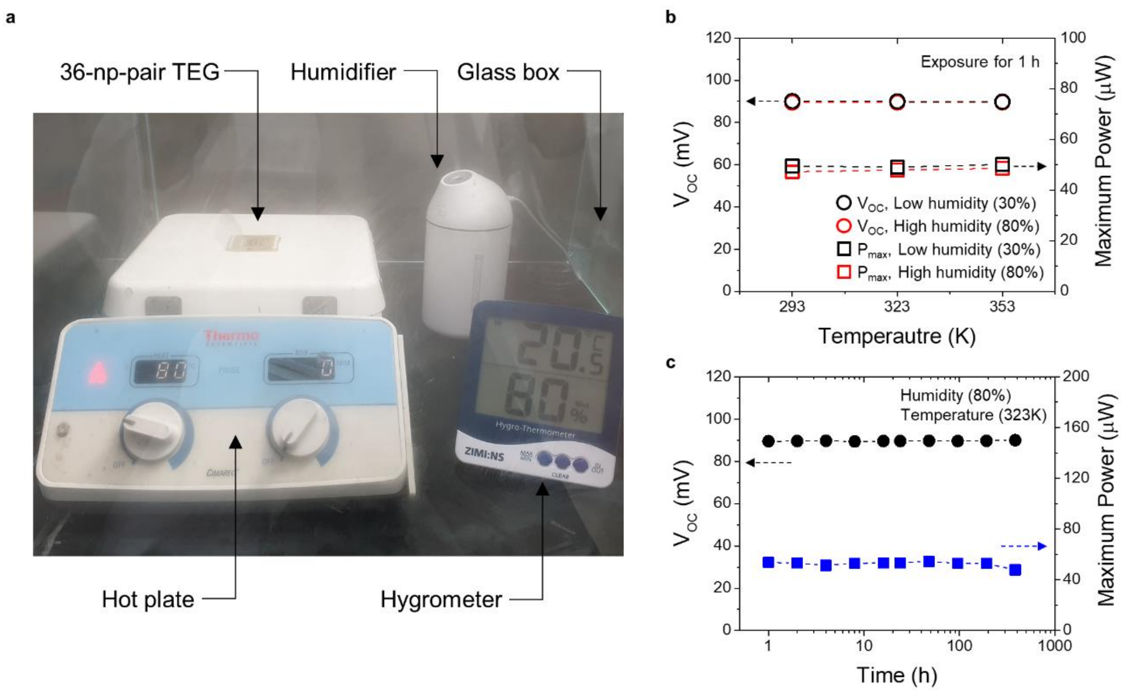

Supplementary Figure 22 | TE performance of the compliant TEG under different humidity and temperature conditions. **a**, Optical image of the experimental setup for investigating the humidity and temperature dependence of the TEG performance. **b** and **c**, Experimentally measured V_{OC} and maximum power of 36-np-pair TEG exposed at the different humidity and temperature conditions (**b**) and the performance at exposure time to high humidity and temperature conditions (**c**) for a given temperature difference of 10 K.

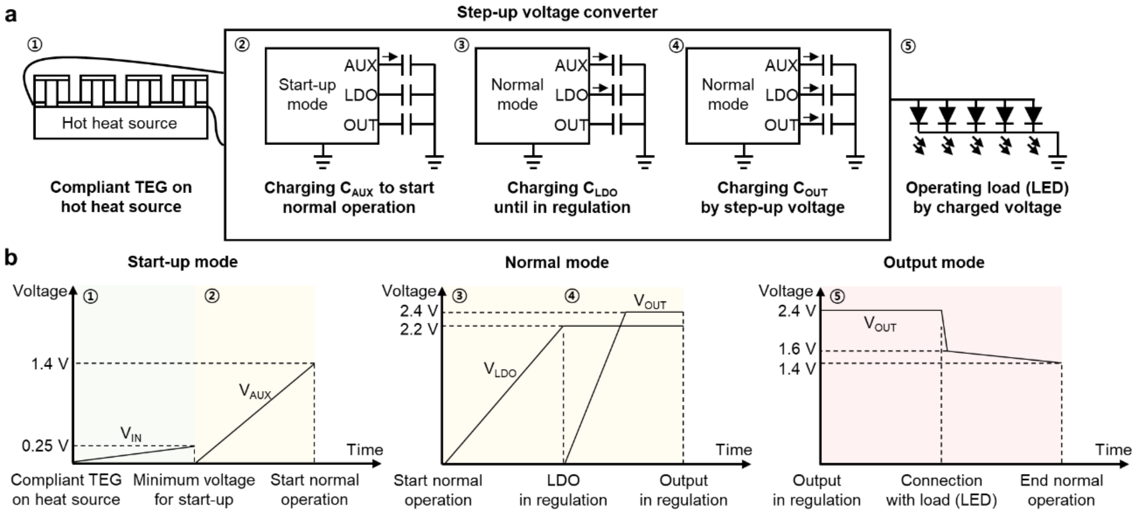

Supplementary Figure 23 | Step-up voltage converter circuit for self-powered wearable applications. a and **b**, Schematic illustration (**a**) and simplified schematic graphs (**b**) showing operation sequence of step-up voltage converter to operate warning system with five light-emitting diodes (LEDs).

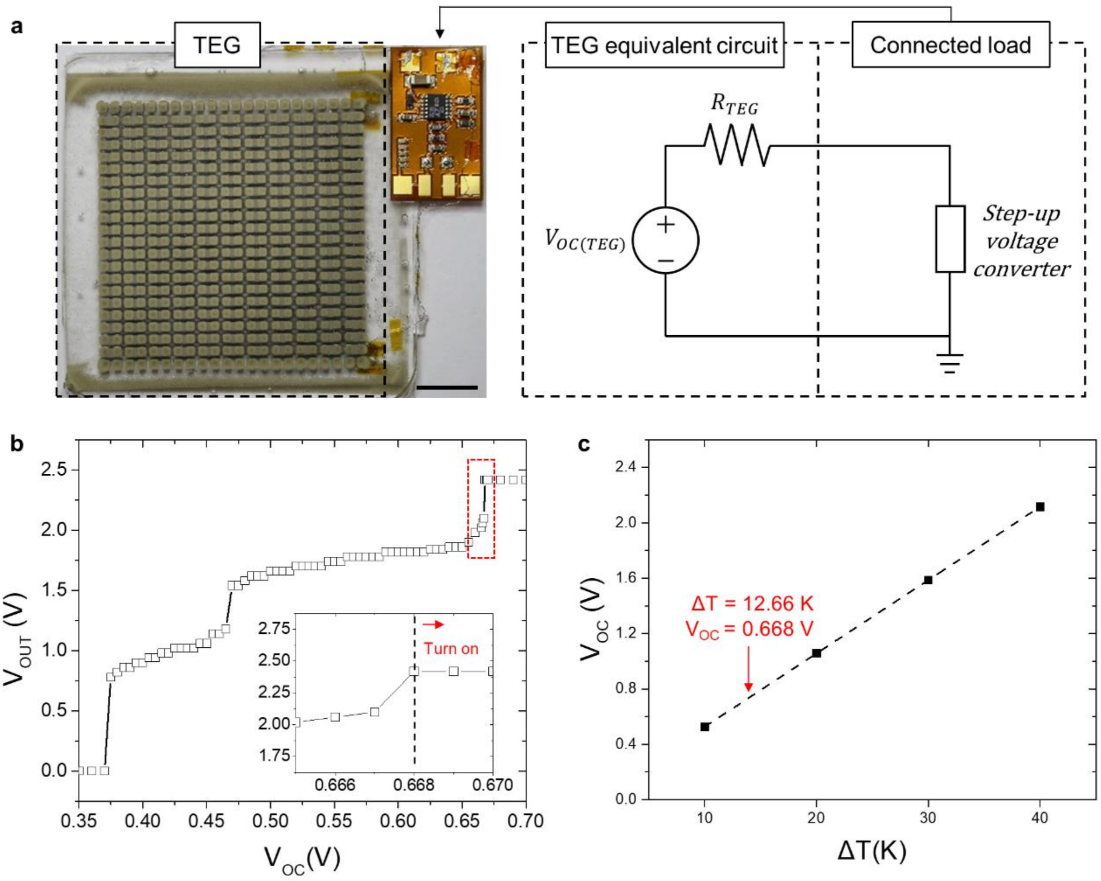

Supplementary Figure 24 | Minimum temperature differences of compliant TEG for turning on LEDs. a, Photograph and an equivalent circuit diagram of 220-np-pair compliant TEG with designed a flexible printed circuit board (f-PCB) with a step-up voltage converter and five mini-LEDs circuit. Scale bar, 1 cm. **b**, Output voltage of step-up voltage converter as a function of input V_{OC}. The inset shows an enlarged view of the part of the output voltage plot when the step-up voltage converter enables LEDs to turn on. **c**, Linear fitted plot of V_{OC} as a function of temperature difference of the TEG to extract minimum temperature difference to turn on the LEDs.

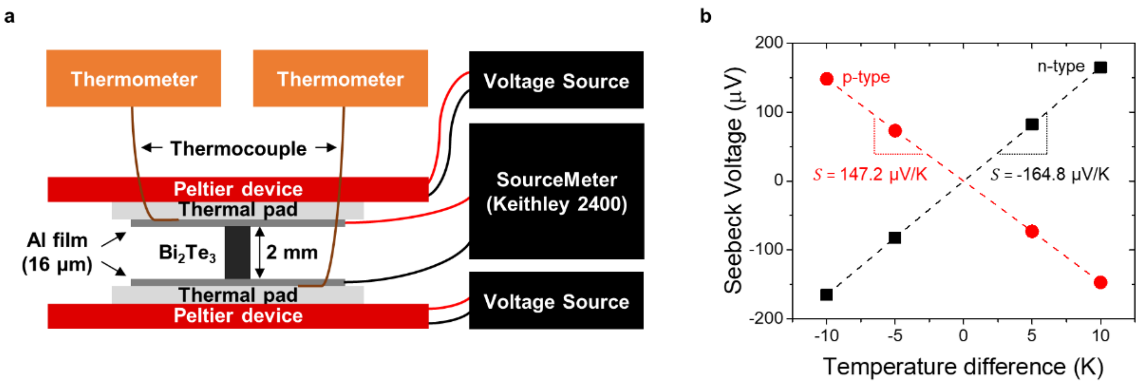

Supplementary Figure 25 | Seebeck coefficient of *p*- and *n*-type Bi_2Te_3 TE leg. a, Schematic illustration of a measurement system for introducing temperature differences and measuring Seebeck voltage of the *p*- and *n*-type Bi_2Te_3 TE leg. **b**, Experimentally measured the Seebeck voltage as a function of temperature difference to extract the Seebeck coefficient of *p*- and *n*-type Bi_2Te_3 TE leg.

Supplementary Tables

Supplementary Table 1 | Experimental parameters of the magnetic self-assembly process for implementing s-HCs.

	Material	Parameter		
**Elastomer matrix**	PDMS (Polydimethylsiloxane)	Mixing ratio (base:curing agent)		10:1
		Viscosity		2.4 Pa s
		Density		0.965 g/cm^3
**Fillers**	Silver-coated nickel (Ag-Ni) particles	Ag concentration		39.9 wt%
		Average size		9.0 μm
		Density		2.776 g/cm^3
**Magnets**	Samarium cobalt	Magnetic flux intensity		1,600 G
**Soft heat conductors**	Ag-Ni particles/PDMS composite	Ag-Ni concentration	Before patterning	25.79 v/v% (50 wt%)
			After patterning /in self-assembled s-HCs	64.98 v/v% (84.23 wt%)

Supplementary Table 2 | TE properties of bismuth telluride (Bi_2Te_3) legs.

Parameter / Material	Dimension *(H×W×D) (mm^3)	Seebeck coefficient (μV/K)	Electrical conductivity (S/m)	**Thermal conductivity (W/(m·K))
p-type	2×1×1	147.2	6.11×10^4	1.8 ~ 2.0
n-type	2×1×1	-164.8	3.41×10^4	1.8 ~ 2.0

* H = Height, W = Width, D = Depth

** Thermal conductivity of each Bi_2Te_3 leg is referred in datasheet from Kyrotherm

Supplementary Table 3 | Comparison of TE performance with previous compliant TEGs. The limitations of previously reported approaches are highlighted in red.

Ref.	Strategy	Interfacing substrate (thermal conductivity, W m^{-1} K^{-1})	Legs	Area (cm^2)	ΔT (K)	V_{OC} (mV)	Normalized Seebeck voltage per unit area (μV K^{-1} cm^{-2})	Normalized power density (μW cm^{-2} K^{-2})	Flexibility	Stretchability
This works	Bi_2Te_3-based TE leg /AgNW electrodes	s-HC (1.4)	72	2.55	40	362	3549	0.203		20%
This works	Bi_2Te_3-based TE leg /AgNW electrodes	s-HC (1.4)	440	16.77	40	2116	3154	0.262		20%
1	BiTe-based TE leg /LM electrodes	Silicone composite (0.28)	8	2.02	20	3	74.4	0.1		20%
2	silicone coils	PDMS (0.16)	128	2.25	19	51.3	1200	2.5E-06		60%
3	Transition metal dichalcogenide nanosheets	PDMS (0.16)	32	10	3	2.4	80	8.1E-05		50%
4	BiTe-based TE film /Cu electrodes	PDMS (0.16)	8	3	50	90	600	**1.52**	20 mm	
5	BiTe-based TE leg /Cu electrodes	PDMS (0.16)	144	16	25	500	1250	**7.648**	5 mm	
6	BiTe-based TE leg /Cu electrodes	PI film	36	12.6	12	48	317.5	0.02083	NA	
7	BiTe-based TE leg /Cu electrodes	PDMS (0.16)	104	11.5	50	37.2	64.5	0.00668	15 mm	
8	BiTe-based TE leg /Cu electrodes	Ecoflex/AlN (0.77)	144	25	6.6	165	1000	**0.576**	20 mm	
9	BiTe-based TE film /PEDOT:PSS hybrid composites	PI film	14	NA	50	85.2	NA	**0.48**	15 mm	
10	BiTE-based TE film /polymer fabric	fabric	24	1.5	20	25	833.3	0.00037	NA	
11	Nanomaterial/fabric/silver foil electrodes	fabric	24	32	35	10	8.93	3.8E-07	NA	
12	PEDOT:PSS/MWCNT-based yarns	fabric	20	36	66	0.8	0.337	1.66E-08	NA	

Supplementary Table 4 | Simulation parameters for finite element analysis (FEA).

Material \ Parameter	Heat capacity (J/(kg·K))	Seebeck coefficient (μV/K)	Electrical conductivity (S/m)	Thermal conductivity (W/(m·K))
s-HC	1460	-	-	1.4
p-Bi_2Te_3	154	147.2	6.11×10^4	1.9
n-Bi_2Te_3	154	-164.8	3.41×10^4	1.9
AgNW	1460	-	10^6	1.0
PDMS	1460	-	2.5×10^{-14}	0.16

Supplementary Table 5 | Resistance of the resistive components in the compliant TEG.

No.	Component	Resistance (Ω)	Numbers in a 72-leg module	Total resistance in a 72-leg module (Ω)	Ratio (%)
(1)	n-type TE leg	0.01713	36	0.6167	2.2
(2)	p-type TE leg	0.01319	36	0.4748	1.7
(3)	AgNW electrode	0.07494	71	5.3209	19.2
(4)	Conductive epoxy junction	0.14748	144	21.2370	76.8
cf.	Cu electrode	0.0008	-	-	-

Supplementary Note

Supplementary Note 1 | Calculation of Ag-Ni particle concentration in the s-HCs. The Ag-Ni particle concentration in the s-HCs needs to be calculated to investigate the heat transfer ability of the s-HCs. Because randomly distributed Ag-Ni particles in a PDMS precursor converged at the iron pillar locations by the patterned magnetic field (see the photograph of the pillar in Supplementary Fig. 3a), the concentration of the Ag-Ni particles in the s-HC areas is much higher than the initial concentration. In particular, all particles outside the pillar areas moved inside the closest pillar areas, forming the same square patterns with the pillar areas (Supplementary Fig. 3b). For the calculation of the Ag-Ni particle concentration in the square pattern, we defined a "unit area" as indicated in red rectangles in Supplementary Fig. 3c and d, and a "patterned area" that corresponds to the area of the iron pillar (blue rectangles in Supplementary Fig. 3c and d). Since the iron pillars are placed with a regular interval, and the Ag-Ni particles were uniformly mixed with a PDMS precursor, the same amount of the particles in each unit area converged at each patterned area for all of the iron pillars. Note that there remained no particles in the area between the patterned areas, as shown in Supplementary Fig. 3d. The Ag-Ni particle concentration in the patterned area is calculated using the initial volume fraction and the ratio between the unit and patterned area. Because the same amount of PDMS and Ag-Ni particles are mixed, the initial weight concentration of the mixture can be described as follows:

$$\frac{M_{\mathrm{AgNi}}}{M_{\mathrm{AgNi}}+M_{\mathrm{PDMS}}} = 50\ \mathrm{wt\%} \qquad (1)$$

where M_{AgNi} and M_{PDMS} are the mass of Ag-Ni particles and PDMS, respectively. The volume ratio can be calculated for the given mass of the Ag-Ni particles and PDMS using the relation between volume and mass as follows:

$$\frac{V_{\mathrm{AgNi}}}{V_{\mathrm{AgNi}}+V_{\mathrm{PDMS}}} = \frac{M_{\mathrm{AgNi}}/D_{\mathrm{AgNi}}}{M_{\mathrm{AgNi}}/D_{\mathrm{AgNi}}+M_{\mathrm{PDMS}}/D_{\mathrm{PDMS}}} = 25.79\% \qquad (2)$$

where V_{AgNi} and V_{PDMS} are the volume and D_{AgNi} (2.776 g/cm^3) and D_{PDMS} (0.965 g/cm^3) are the densities for Ag-Ni particles and PDMS, respectively. After the magnetic self-assembly, as the Ag-Ni particles converge from the unit area to the patterned area, the increased volume concentration is calculated by considering the volume ratio between the unit and patterned areas. Because the thickness is same before and after magnetic self-assembly, only the areas of the unit and patterned area need to be considered. The increased volume concentration can be described as follows:

$$F = \frac{V_{\mathrm{AgNi}}}{V_{\mathrm{AgNi}}+V_{\mathrm{PDMS}}} \times \frac{A_{\mathrm{Unit}}}{A_{\mathrm{Pattern}}} = 64.98\% \qquad (3)$$

where A_{Unit} (4 mm^2) and A_{Pattern} (1.588 mm^2) are the areas of the unit and patterned area. The weight concentration after magnetic self-assembly can be expressed using equation (3) and densities of the materials as follows:

$$\frac{D_{\mathrm{AgNi}} \cdot F}{D_{\mathrm{AgNi}} \cdot F + D_{\mathrm{PDMS}} \cdot (1-F)} = 84.23\ \mathrm{wt\%} \qquad (4)$$

The calculated concentration in equation (4) is much higher than the initial concentration of 50 wt%. Together with a chain-like morphology of self-assembled Ag-Ni particles, this increased particle concentration in the patterned area plays an important role in enhancing the heat transfer ability of s-HCs.

Supplementary References

1. Jeong, S. H. et al. Stretchable Thermoelectric Generators Metallized with Liquid Alloy. *ACS Appl. Mater. Interfaces* **9**, 15791-15797 (2017).

2. Nan, K. et al. Compliant and stretchable thermoelectric coils for energy harvesting in miniature flexible devices. *Sci. Adv.* **4**, eaau5849 (2018).

3. Oh, J. Y. et al. Chemically exfoliated transition metal dichalcogenide nanosheet-based wearable thermoelectric generators. *Energy Environ. Sci.* **9**, 1696-1705 (2016).

4. Kim, S. J., We, J. H. & Cho, B. J. A wearable thermoelectric generator fabricated on a glass fabric. *Energy Environ. Sci.* **7**, 1959-1965 (2014).

5. Kim, S. J. et al. High-Performance Flexible Thermoelectric Power Generator Using Laser Multiscanning Lift-Off Process. *ACS Nano* **10**, 10851-10857 (2016).

6. Liu, H., Wang, Y., Mei, D., Shi. Y. & Chen, Z. Design of a Wearable Thermoelectric Generator for Harvesting Human Body Energy. *Wear. Sens. Robots* **399**, 55-66 (2016).

7. Wang, Y., Shi, Y., Mei, D. & Chen, Z. Wearable thermoelectric generator to harvest body heat for powering a miniaturized accelerometer. *Appl. Energy* **215**, 690-698 (2018).

8. Hong, S. et al. Wearable thermoelectrics for personalized thermoregulation. *Sci. Adv.* **5**, eaaw0536 (2019).

9. We, J. H., Kim, S. J. & Cho, B. J. Hybrid composite of screen-printed inorganic thermoelectric film and organic conducting polymer for flexible thermoelectric power generator. *Energy* **73**, 506-512 (2014).

10. Kim, M.-K., Kim, M.-S, Lee, S., Kim, C. & Kim, Y.-J. Wearable thermoelectric generator for harvesting human body heat energy. *Smart Mater. Struct.* **23**, 105002 (2014).

11. Lu, Z., Zhang, H., Mao, C. & Li, C. M. Silk fabric-based wearable thermoelectric generator for energy harvesting from the human body. *Appl. Energy* **164**, 57-63 (2016).

12. Wu, Q. & Hu, J. A novel design for a wearable thermoelectric generator based on 3D fabric structure. *Smart Mater. Struct.* **26**, 045037 (2017).

13. Suarez, F. et al. Flexible thermoelectric generator using bulk legs and liquid metal interconnects for wearable electronics. *Appl. Energy* **202,** 736-745 (2017).

14. Jo, S. E., Kim, M. K., Kim, M. S. & Kim, Y. J. Flexible thermoelectric generator for human body heat energy harvesting. *Electron. Lett.* **48,** 1015-1017 (2012).

15. Kim, C. S. et al. Structural design of a flexible thermoelectric power generator for wearable applications. *Appl. Energy* **214,** 131-138 (2018).
