# Supplementary material for: High-performance compliant thermoelectric generators with magnetically self-assembled soft heat conductors for self-powered wearable electronics
Source: Nat Commun. 2020 Nov 23;11:5948. doi: 10.1038/s41467-020-19756-z (PMC7684283; doi:10.1038/s41467-020-19756-z)
Supplement: Supplementary file 2 — Description of Additional Supplementary Files [file 41467_2020_19756_MOESM2_ESM.pdf]

Title: Supplementary Movie 1

Description: Fabrication procedures for the compliant TEG. Whole fabrication processes for realizing a 36-np-pair compliant TEG including the details of the magnetic self-assembly of s-HCs and automatic integration of TE legs.

Title: Supplementary Movie 2

Description: Hot surface warning gloves with a self-powered LED system. Demonstration of hot surface warning gloves by integrating the self-powered LED system driven by a 220-np-pair compliant TEG. The conformal contact between the compliant TEG and 3D surfaces (hot bottle in this work) resulted in a bright 'H' sign due to the LEDs being turned on, without the assistance of external power.
